# Urea derivative MTU improves stress tolerance and yield in wheat by promoting cyclic electron flow around PSI

**DOI:** 10.3389/fpls.2023.1131326

**Published:** 2023-03-07

**Authors:** Jaroslav Nisler, Zuzana Kučerová, Radoslav Koprna, Roman Sobotka, Jana Slivková, Stephen Rossall, Martina Špundová, Alexandra Husičková, Jan Pilný, Danuše Tarkowská, Ondřej Novák, Mária Škrabišová, Miroslav Strnad

**Affiliations:** ^1^ Isotope Laboratory, Institute of Experimental Botany, Czech Academy of Sciences, Prague, Czechia; ^2^ Department of Biophysics, Faculty of Science, Palacký University, Olomouc, Czechia; ^3^ Department of Chemical Biology, Faculty of Science, Palacký University, Olomouc, Czechia; ^4^ Centre Algatech, Institute of Microbiology, Czech Academy of Sciences, Třeboň, Czechia; ^5^ Department of Biochemistry, Faculty of Science, Palacký University, Olomouc, Czechia; ^6^ School of Biosciences, Nottingham University, Loughborough, United Kingdom; ^7^ Laboratory of Growth Regulators, Institute of Experimental Botany of the Czech Academy of Sciences & Palacký University, Olomouc, Czechia

**Keywords:** cyclic electron flow, drought stress, heat stress, 1-(2-methoxyethyl)-3-(1,2,3-thiadiazol-5yl)urea, MTU, photosystem I, stress tolerance, wheat

## Abstract

Increasing crop productivity under optimal conditions and mitigating yield losses under stressful conditions is a major challenge in contemporary agriculture. We have recently identified an effective anti-senescence compound (MTU, [1-(2-methoxyethyl)-3-(1,2,3-thiadiazol-5yl)urea]) in *in vitro* studies. Here, we show that MTU delayed both age- and stress-induced senescence of wheat plants (*Triticum aestivum* L.) by enhancing the abundance of PSI supercomplex with LHCa antennae (PSI-LHCa) and promoting the cyclic electron flow (CEF) around PSI. We suppose that this rarely-observed phenomenon blocks the disintegration of photosynthetic apparatus and maintains its activity as was reflected by the faster growth rate of wheat in optimal conditions and under drought and heat stress. Our multiyear field trial analysis further shows that the treatment with 0.4 g ha^-1^ of MTU enhanced average grain yields of field-grown wheat and barley (*Hordeum vulgare* L.) by 5-8%. Interestingly, the analysis of gene expression and hormone profiling confirms that MTU acts without the involvement of cytokinins or other phytohormones. Moreover, MTU appears to be the only chemical reported to date to affect PSI stability and activity. Our results indicate a central role of PSI and CEF in the onset of senescence with implications in yield management at least for cereal species.

## Highlights

The urea derivative MTU increases the abundance and activity of PSI, thereby delaying age- and stress-induced senescence of wheat leaves, thus accelerating its growth and improving yield.

## Introduction

The primary challenge in agricultural science today is to develop technologies that will increase food production and the sustainability of agriculture, especially under environmentally limiting conditions such as drought, heat, and salinity. In those regions where water availability is limiting, crop yields are much lower, and there is great interest in, and potential for, making improvements (Munns and Tester, 2008). For example, while the yield of wheat in the UK has doubled from 4 to 8 tons per hectare over the past 50 years, the average yield in Australia has only increased from 1 to 1.5 tons per hectare (Richards et al., 2010). Low productivity of crops under adverse conditions is a result of plant acclimation to stress. The acclimation can be characterized by specific morphogenic and developmental responses, such as inhibition of shoot growth and acceleration of senescence processes (Potters et al., 2007). This stress-induced senescence causes reductions in grain filling and biomass yield due to leaf yellowing and disrupted photosynthesis. This is a major worldwide agro-economic problem (Reguera et al., 2012).

Wheat belongs to crops that undergo sequential leaf senescence, therefore in addition to stress, the development and demise of individual wheat leaves are driven by age (i.e. developmental = natural leaf senescence). Different factors, such as different stressors or age, induce senescence through different signaling molecules/phytohormones and pathways. For example, drought, salinity, and low temperatures were shown to cause senescence in Arabidopsis through the elevation of abscisic acid (ABA) (Yang et al., 2011), which seems to be a messenger when sugar level decreases as a result of a decline in photosynthetic activity (e.g. Asad et al., 2021). Shadow or complete darkness and wounding trigger senescence through an ethylene-response pathway (Abeles et al., 1988; Ferrante et al., 2002; Buchanan-Wollaston et al., 2005). The salicylic acid response pathway plays a role in the natural senescence of Arabidopsis leaves (Buchanan-Wollaston et al., 2005). On the other hand, our current knowledge, based also on the modern multi-omics approaches, indicates that the senescence process itself exhibits a certain pattern at the cellular and molecular levels regardless of the origin of its initial promoting factor (Gan and Amasino, 1997; Gan, 2003; Guo and Gan, 2012; reviewed in Kim et al., 2016). In other words, once leaf senescence has started, it always proceeds in a similar way. Chloroplasts are broken down first, because they contain the majority of the nitrogen in a leaf, while the nucleus remains intact to control the recycling process (Makino and Osmond, 1991; Domínguez and Cejudo, 2021). Macroscopically, the leaf yellowing is the first visible sign of leaf senescence and results from the preferential degradation of the chlorophyll but not the carotenoids, which are yellow-red pigments (reviewed by Matile et al., 1999). In Arabidopsis, the visible yellowing is widely used to stage the progression of senescence, which reproducibly correlate with other biochemical changes that occur during leaf senescence (e.g. Lohman et al., 1994; Jing et al., 2002). Chlorophyll degradation is tightly linked to the breakdown of chlorophyll-binding proteins in both photosystems (Hörtensteiner, 2006; reviewed by Domínguez and Cejudo, 2021). Although intensively investigated, the sequence of events taking place during the dismantling of PSI and PSII has not been fully elucidated and might vary among species. In cereal crops, the chlorophyll-protein complexes of PSI seem to be degraded preferentially (faster) during senescence (Miersch et al., 2000; Tang et al., 2005).

We recently described a group of urea-derived compounds with exceptionally high anti-senescence activity (Nisler et al., 2018). Among these urea derivatives, 1-(2-methoxyethyl)-3-(1,2,3-thiadiazol-5yl)urea (MTU, formerly ASES) proved to be the most effective at retarding chlorophyll degradation in detached wheat leaves placed in darkness, surpassing also the activity of known cytokinins. A comparison of the effect of MTU and a related strong cytokinin – thidiazuron (TDZ) – on these leaves showed that MTU-treated leaves were greener, produced less ethylene, and showed lower oxidative damage than TDZ-treated leaves. Unlike TDZ, the treatment with MTU induced the accumulation of dimeric PSII and PSII-Light-harvesting complexes II supercomplexes (PSII-LHCII). Interestingly and in distinct contrast to MTU performance in wheat leaf senescence assay, we found that MTU exhibited only weak cytokinin activity in molecular and classical cytokinin bioassays. Taken together, our previous *in vitro* analyses introduced MTU as a highly potent inhibitor of senescence, that appears to act through a cytokinin-independent mechanism (Nisler et al., 2018). However, the effect of MTU on living plants and its agronomical potential has not been sufficiently studied.

In the present study, we prove that the positive effect of MTU on stress tolerance in wheat is not mediated by cytokinins or cytokinin signaling, demonstrating that MTU utilizes another mechanism to inhibit senescence in plants. Here we show that this mechanism is associated with enhanced stability of PSI and its ability to maintain cyclic electron flow (CEF). In order to define the possibilities of practical use of the MTU, we characterized its effect on the growth and yield of wheat and barley plants under optimal, stress, and field conditions. Thus, our results not only confirm the claim of a role for CEF in stress tolerance but provide an important missing link between CEF intensity and grain yield at least in cereal species.

## Materials and methods

### Chemicals

1-(2-Methoxyethyl)-3-(1,2,3-thiadiazol-5yl)urea (MTU, CAS Number: 1850376-35-0) was synthesized as described previously (Nisler et al., 2018). *Trans*-zeatin (CAS Number: 1637-39-4) and thidiazuron (CAS Number: 51707-55-2) were purchased from Olchemim (Czech Republic).

### SPAD and weight analysis of 20-48-day old plants

**Figure 1:**
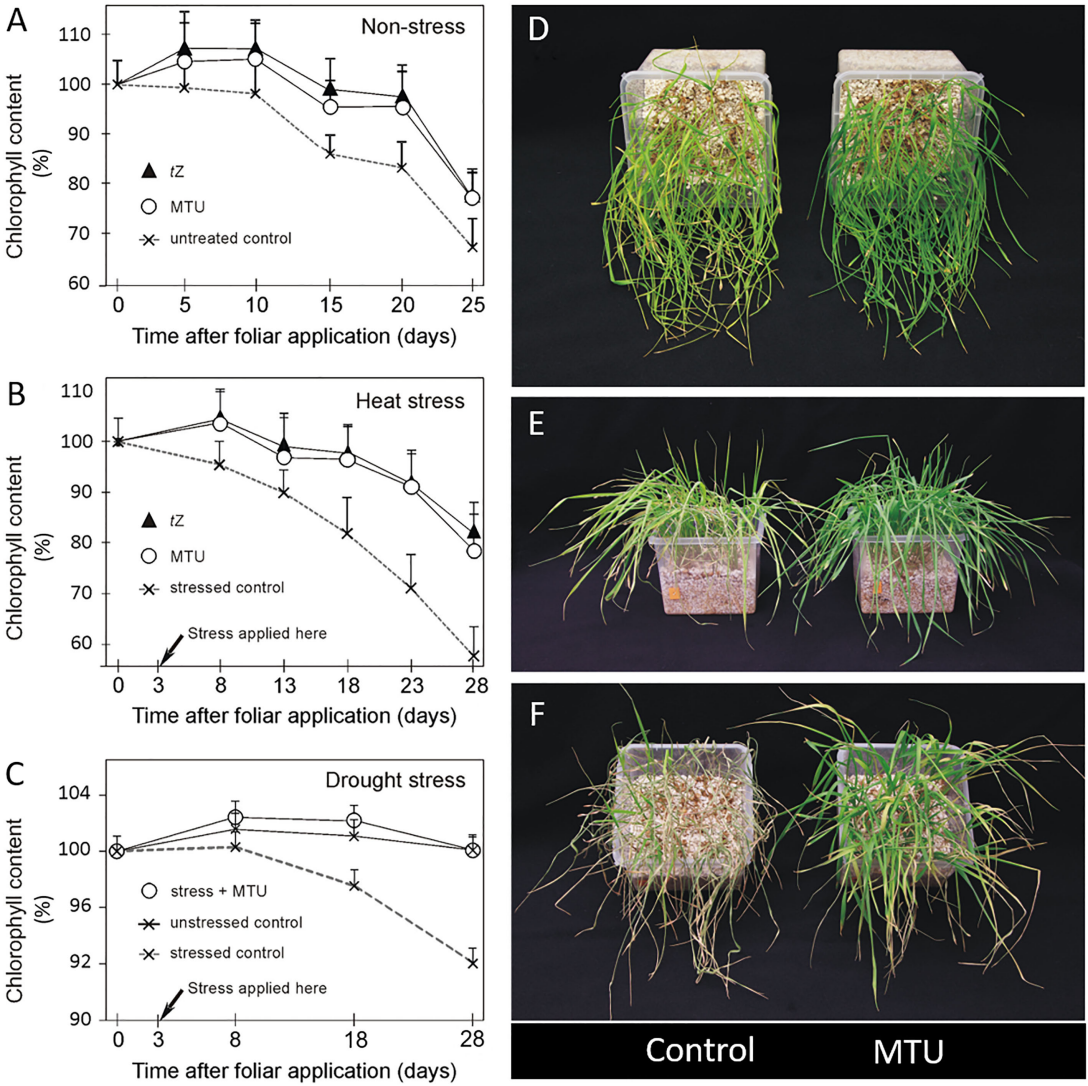
The effect of MTU (and *t*Z) on chlorophyll content in wheat. **(A-C)** The 20-day-old plants were treated with water (untreated control), 1-(2-methoxy-ethyl)-3-1,2,3-thiadiazol-5-yl urea (MTU), or *trans*-zeatin (*t*Z) and grown another 25/28 days under optimal conditions **(A)**, heat stress conditions **(B)**, and drought stress conditions **(C)**. Relative chlorophyll content was assessed on third leaves by SPAD meter. Values are means + SD of ten biological replicates and are expressed in % of chlorophyll content before application (day 0). Weight parameters of these plants (harvested 45 or 48 days after planting) are shown in **Table 1**. **(D)** 21-Day-old plants held for the last seven days without water. **(E)** The same 23-day-old plants held for 8 days without water and photographed 24 hours after re-watering. **(F)** 25-Day-old plants held for the last 10 days without water, photographed 24 hours after re-watering.

This description of the experiment is related to the results in **Table 1**; **Figures 1A–C** and **Supplemental Figures S1**, **S2**. Winter wheat (var. Skyfall) was raised in peat-based module compost until the second leaf had emerged. The plants were then transplanted into deep pots (diameter 120 mm, depth 180 mm) filled with 10 mm washed expanded clay pellets (Hydroleca, GB) and maintained in a hydroponic system by daily irrigation with 20:8:20 NPK fertilizer containing trace elements (OMEX, GB). The plants were grown in a greenhouse under natural light in the summer months in the UK with day venting at 18°C, and night heating at 12°C. The plants were grown-on to the four-leaf stage (20-day-old). Then, water (control), 10 μM *t*Z, or 10 μM MTU was sprayed on foliage in a volume of water equivalent to 200 L ha^-1^. Twenty replicate plants were set up for each interaction. Three days after spraying, half of the plant population was transferred to a hot greenhouse maintained at a constant 30°C for 25 days to impose heat stress. All treated plants were randomly arranged in trays. Relative chlorophyll content was assessed on third leaves using a SPAD meter. In case of plants grown under optimal conditions, the plants were harvested 25 days after compound treatment. In case of plants grown under heat conditions, the plants were harvested 28 days after compound treatment, allowing them to have three days between compound and stress application. After harvest, the roots and shoots were washed in water, and dried at 70°C for 2 days, after which their dry weights were determined. For each interaction, two visually-assessed representative root systems were selected and photographed alongside the ‘average’ untreated controls.

**Table 1:** Weight parameters and chlorophyll content in 45 or 48-days-old wheat plants.

(A)	Non-stressed wheat*									
	RDW(g)	%	SDW(g)	%	TDW(g)	%	SPAD	%	CCf	%
control	0.80 ± 0.06	100	1.52 ± 0.17	100	2.32 ± 0.16	100	33.1 ± 2.8	100	50.3 ± 7.0	100
*t*Z	1.00 ± 0.1	**125 *c***	1.65 ± 0.17	**109**	2.65 ± 0.21	**114 *c***	38.0 ± 2.7	**115 *c***	62.7 ± 8.1	**125 *b***
MTU	0.99 ± 0.14	**123 *c***	1.66 ± 0.18	**110 *a***	2.65 ± 0.23	**114 *c***	37.8 ± 2.7	**114 *c***	63.0 ± 9.5	**125 *b***
Heat-stressed wheat										
control	0.92 ± 0.15	100	1.37 ± 0.09	100	2.30 ± 0.18	100	28.2 ± 2.9	100	38.7 ± 5.1	100
*t*Z	1.14 ± 0.19	**124 *b***	1.41 ± 0.12	**103**	2.55 ± 0.26	**111 *b***	40.6 ± 2.8	**144 *c***	57.1 ± 5.4	**147 *c***
MTU	1.27± 0.07	**137 *c***	1.56 ± 0.13	**113 *c, a***	2.82 ± 0.18	**123 *c, a***	38.6 ± 3.6	**137 *c***	60.0 ± 6.3	**155 *c***
(B)	Non-stressed wheat									
	RDW(g)	%	SDW(g)	%	TDW(g)	%	SPAD	%	CCf	%
Control	0.91 ± 0.08	100	1.61 ± 0.16	100	2.52 ± 0.22	100	44.5 ± 2.7	100	71.8 ± 7.5	100
MTU	1.01 ± 0.14	**112**	1.81 ± 0.17	**112 *a***	2.82 ± 0.15	**112 *b***	46.7 ± 3.2	**105**	84.7 ± 10.3	**118 *b***
Drought-stressed wheat										
Control	0.75 ± 0.07	100	1.22 ± 0.14	100	1.97 ± 0.14	100	41.4 ± 3.4	100	50.6 ± 7.4	100
MTU	1.06 ± 0.12	**142 *c***	1.34 ± 0.11	**109**	2.40 ± 0.19	**122 *c***	44.0 ± 2.8	**106**	58.9 ± 6.3	**116 *a***

MTU, 1-(2-methoxyethyl)-3-(1,2,3-thiadiazol-5yl)urea; *t*Z, *trans*-zeatin; RDW, root dry weight; SDW, shoot dry weight; TDW, total dry weight; SPAD, chlorophyll content determined by SPAD meter (in absolute units); CCf, chlorophyll coefficient per plant (equal to SDW x SPAD); * Plants were 45-days-old in a day of harvest.

The 20-day-old plants were sprayed with water (control), MTU or *t*Z and then cultivated for 25/28 days under optimal or heat (A) and drought (B) stress conditions in two independent experiments. Values are means (± SD) of results for ten biological replicates. Statistically significant differences between control and treatments (black letters) and between treatments (red letters) identified by the two‐tailed Student’s t‐test are indicated by the letters a, b or c, corresponding to P-values of 0.05 > P > 0.01, 0.01 > P > 0.001, and P < 0.001, respectively. % - means a percentage of control. Chlorophyll content in the third leaves of these plants was also determined in more time points after compound/stress application (see **Figures 1 A–C**). Bold text show percentage change in the value.

The drought stress experiment was performed in the same way. Stressed plants were grown in the same conditions as control plants, but for 28 days received limited water supply, when showing signs of wilting. Soil relative water content was maintained between 60 and 70%. All plants were harvested 28 days after compound application (48 days after planting).

### Photo documentation

This description of the experiment is related to the results in **Figures 1D–F**. Seeds of winter wheat (cv. Balitus) were sown in pots (0.5 L) with perlite and Hoagland’s solution and grown in a walk-in growth chamber with a 14/10 (light/dark) photoperiod, a light intensity of 150 - 200 µmol photons m^−2^ s^−1^, 60% humidity, and a temperature of 19–24°C. Plants were gently watered with tap water such that all containers had equal weights. 7-Day-old plants were sprayed with 0.01% DMSO in water (control) or 10 μM MTU (5 mL/pot) and left to grow another 7 days before watering was halted (**Figures 1D–F**). The relative chlorophyll content in whole plants from **Figure 1D** was determined spectrophotometrically at 664 nm using a methanol extract.

### Pigments analysis in 5-23-day-old plants

**Figure 2:**
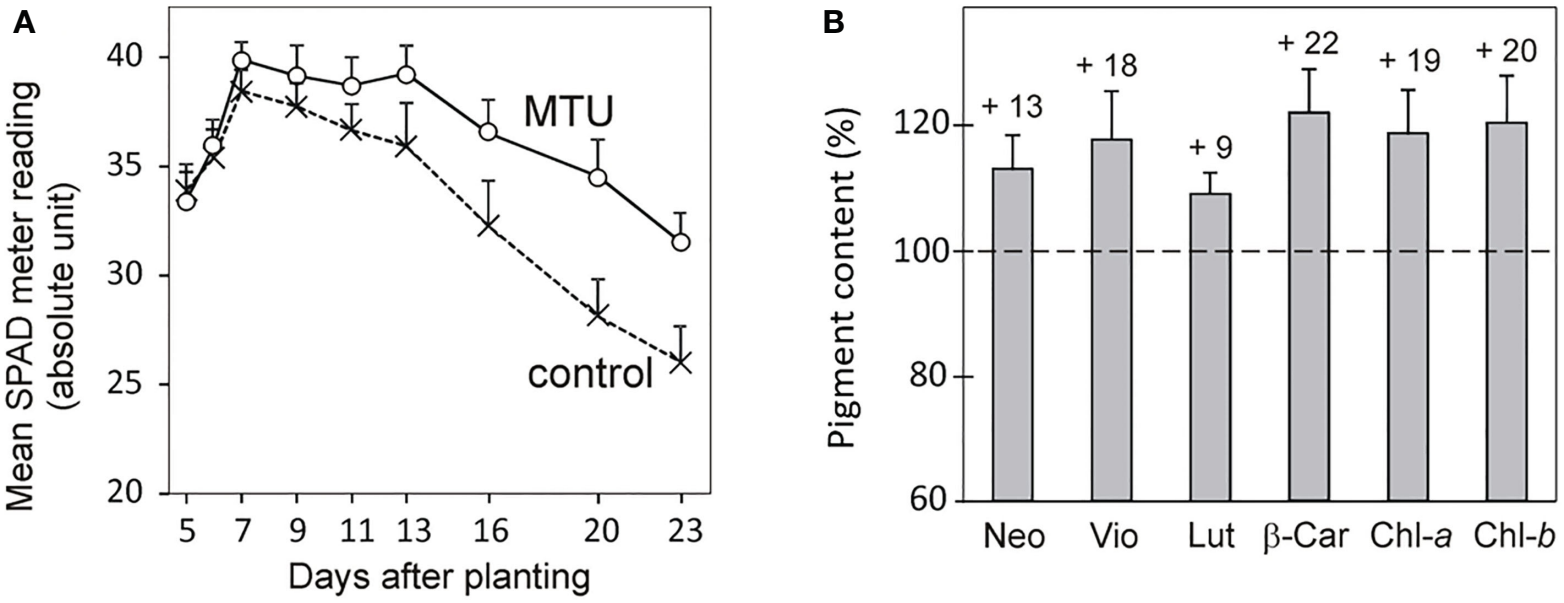
The pigment content in wheat seedlings treated by MTU. **(A)** The chlorophyll content in the first leaves of wheat. Values are means ± SD of 15 biological replicates. All differences from day 7 onwards are statistically significant at *P* < 0.003 based on two‐tailed Student’s *t*‐tests. **(B)** Content of photosynthetic pigments in shoots of 20-day-old wheat plants treated with MTU relative to untreated control plants (dashed line). Values are means ± SD of seven biological replicates. All differences are statistically significant based on two‐tailed Student’s *t*‐tests at *P* < 0.02. Numbers above each column show percentage increases. Neo, neoxanthin; Vio, violaxanthin; Lut, lutein; β-Car, β-carotene; Chl-*a*, chlorophyll *a*; Chl-*b*, chlorophyll *b*.

This description of the experiment is related to the results in **Figure 2**. Seeds of spring wheat (cv. Aranka) were sown into 0.5 L pots (approximately 25 seeds per pot) with perlite and Hoagland’s solution (control) or Hoagland’s solution containing MTU (final concentration 75 nM). In this case, MTU was applied to the root system so that the effect of MTU on leaf chlorophyll content could be assessed since the first leaf emergence. Plants were grown in a greenhouse under natural light supported by artificial light when the intensity of natural light fall below 100 µmol m^-2^ s^-1^ (16h per day). The day temperature was 22-25°C, and night temperature was 16-22°C. Plants were gently watered with tap water to equal weights. Relative chlorophyll content (SPAD values) in the first emerging leaves (n=15 per treatment) were obtained by regular measuring the first leaf within 3.0 cm of the leaf tip using a SPAD-502Plus chlorophyll meter (Konica Minolta, Japan) (**Figure 2A**). For quantitative pigment content analysis (**Figure 2B**), shoots of 20-day-old plants (n=7 per treatment) were lyophilized, cut into small pieces (3x3 mm) and mixed with 250 μL of glass beads (100–200 μM in diameter). Pigments were extracted by adding 1 mL of methanol and bead-beating for 10 s in a Mini-Beadbeater (Biospec, USA). After collecting the supernatant, the extraction was repeated. The supernatants were then pooled and the content of pigments in the samples was determined by HPLC (Pazderník et al., 2019).

### Evaluation of leaf water potential and photosynthetic parameters

**Figure 3:**
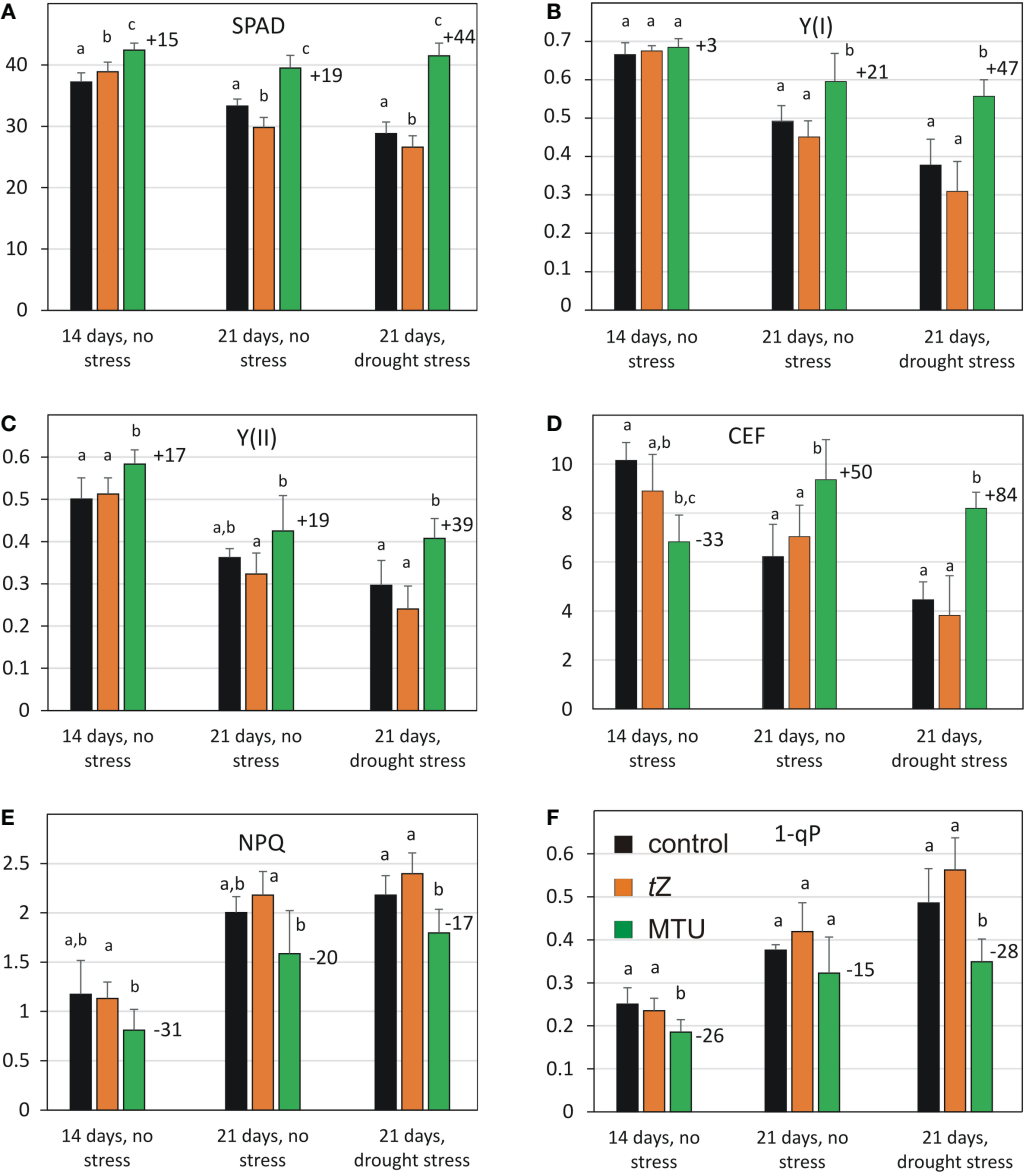
Comparison of chlorophyll content **(A)** and photosynthetic parameters **(B-F)** of wheat during age-related and drought-induced senescence (after 7 days of drought, SRWC 50%). The parameters of untreated plants were compared to those treated with *trans*-zeatin (*t*Z) or 1-(2-methoxy-ethyl)-3-1,2,3-thiadiazol-5-yl urea (MTU). Values are means ± SD of 5 biological replicates. Different letters indicate statistically significant differences at *P* < 0.05 based on the two‐tailed Student’s t‐test. The numbers above the columns show the percentage change in the parameter of MTU-treated plants relative to untreated control. Legend in 3F applies to all Figures. The number of days below the columns indicates the age of the plants. SPAD – relative chlorophyll content analyzed by Soil Plant Analysis Development (SPAD) chlorophyll meter; Y(I), Y(II) – effective quantum yields of PSI and PSII, respectively; CEF, cyclic electron flow; NPQ, non-photochemical quenching; 1-qP – a fraction of reduced PQ pool.

This description of the experiment is related to the results in **Figures 3**, **S3**-**S8**. Spring wheat (cv. Aranka) was cultivated in a greenhouse in conditions as described in the case of pigment analysis (above). Treated plants were cultivated in a presence of MTU or *trans*-zeatin (final concentration 75 nM at the beginning of the experiment). Plants exposed to drought stress were gently watered only until day 14 after planting. On day 21, when the SRWC was around 50%, leaf water potential and photosynthetic parameters were measured. Then plants were harvested and shoot dry weight was determined after 24 h of oven drying at 70°C.

Leaf water potential was measured using a C-52 psychrometric chamber and HR-33T Dew Point Microvoltmeter (Wescor, USA) on segments detached from the primary leaves after 60 min of equilibration in the sample chamber.

Parameters reflecting PSII and PSI functions were measured simultaneously on pre-darkened (for 25 min) intact leaves using a Dual-PAM100 measuring system (Heinz Walz, Effeltrich, Germany) during exposure to red actinic light (125 µmol photons m^–2^ s^–1^) for 11 min and using 300 ms saturating red light pulses (10,000 µmol photons m^–2^ s^–1^). The effective quantum yield of PSII photochemistry in light-adapted state Y(II) was calculated as Y(II)= (F_M_´– F)/F_M_´ (F_M_´ is the maximum chlorophyll fluorescence intensity at a light-adapted state and F is related to chlorophyll fluorescence level at the state induced by the actinic light). The effective quantum yield of PSI photochemistry in light-adapted state Y(I) was calculated using the Dual-PAM100 software according to Klughammer and Schreiber (2008). Cyclic electron flow (CEF) around PSI was calculated as a difference between electron transport rate through PSII and PSI (ETR-II and ETR-I), which are directly related to Y(II) a Y(I), respectively (ETR-II = PAR∙Y(II)∙ 0.84∙0.5, ETR-I = PAR∙Y(I)∙ 0.84∙0.5, where PAR is the irradiation at 400–700 nm and the constants represent the assumed average leaf absorptance of PAR and the fraction of the light absorbed by given photosystem). Non-photochemical quenching was calculated as (F_M_ – F_M_´)/F_M_´) (F_M_ is the maximum chlorophyll fluorescence intensity at a dark-adapted state).

The fraction of reduced PQ pool in thylakoid membranes was evaluated as 1–qP, where qP is the coefficient of photochemical quenching calculated as (F_M_´– F)/F_M_´ – F_0_´) (F_0_´ is the minimum chlorophyll fluorescence intensity at a light-adapted state).

Wheat leaf senescence assay with *t*Z (**Supplemental Figure S9**) was performed as described previously (Kučerová et al., 2020).

### Preparation of thylakoid membranes and two-dimensional electrophoresis

**Figure 4:**
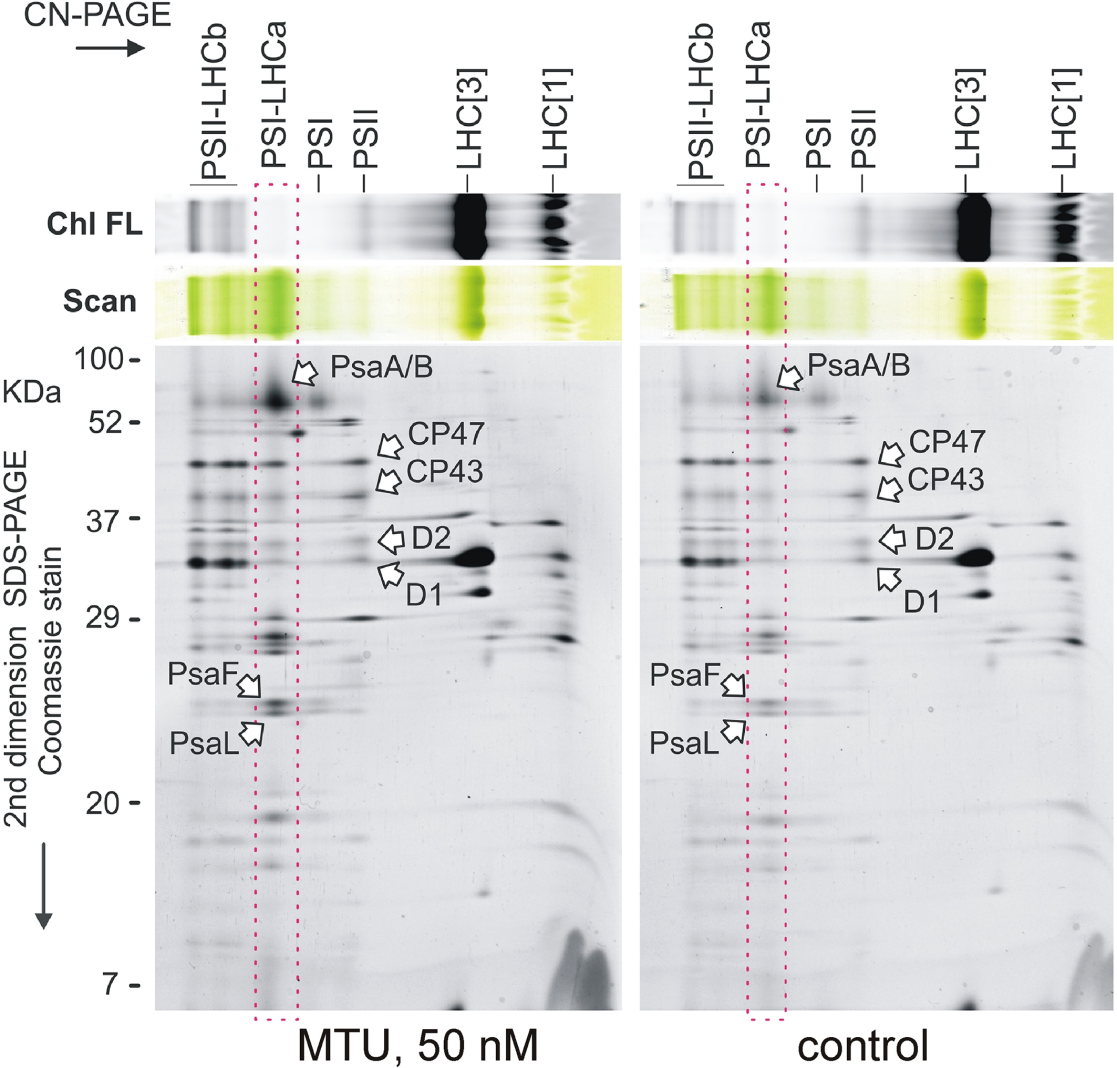
Two-dimensional gel electrophoresis of chloroplast membrane complexes isolated from five 20-day-old wheat plants treated with MTU. Solubilized membranes were separated by clear-native electrophoresis (CN-PAGE) and all samples were loaded based on their chlorophyll content per dry weight. Pigmented complexes were detected in the first dimension by their color (Scan) and chlorophyll fluorescence (Chl FL). The obtained protein complexes were further separated in the second dimension on a denaturing SDS gel. PSII-LHCb, PSII supercomplexes; PSI-LHCa, Photosystem I associated with LHCa antennae; PSI, Photosystem I; PSII, Photosystem II; LHC[1] and LHC[3], monomer and trimeric light-harvesting complexes II; CP47, CP43, D2, and D1 are core subunits of PSII; PsaA/B/F/L are subunits of PSI.

This description of the experiment is related to the results in **Figure 4**. Winter wheat (cv. Balitus) was cultivated in a greenhouse as described in the case of pigment analysis. Treated plants contained 50 nM MTU in Hoagland’s solution. Shoots of 20-day-old wheat plants were collected from five plants per treatment (control and 50 nM MTU). Preparation of thylakoid membranes and two-dimensional electrophoresis was performed as described previously (Nisler et al., 2018). Separated protein spots were identified based on previously published 2D gels of the thylakoid membrane complexes (Aro et al., 2005; Pagliano et al., 2012).

### Phytohormone analysis

This description of the experiment is related to the results in **Table 2** and **Supplemental Tables S1**-**S3**. Winter wheat (cv. Balitus) was cultivated in a walk-in growth chamber as described in the case of photo documentation. 19-Day-old wheat plants were sprayed with 0.01% DMSO in water (control), or 10 μM MTU. Silwet (0.01%) was used as a surfactant in all variants. Starting on day 20, plants were left without water for 5 days (SRWC was 72%), after which plant samples were collected and analyzed. For the analysis of endogenous cytokinins, IAA, and ABA four plants were used per treatment and each sample was analyzed in two replicates. Extraction and purification were performed as described by Dobrev and Kamínek (2002) with minor modifications (Nisler et al., 2018). The levels of cytokinins and IAA were quantified by ultra-high-performance liquid chromatography–electrospray coupled to the tandem mass spectrometry using stable isotope-labeled internal standards as described by Novák et al. (2008). Levels of ABA and GAs were analyzed as described by Floková et al. (2014) and Urbanová et al. (2013), respectively. BRs were extracted, purified, and analyzed as described by Tarkowská et al. (2016). For the analysis of GAs and BRs, three samples (each containing equal quantities of material from two plants) were used per treatment and each sample was analyzed in two technical replicates.

**Table 2:** Phytohormone levels in 25-day-old intact wheat shoots after 5 days of drought treatment (SRWC was 72%).

Hormone	Watered control			Drought control				Drought + MTU				
**ABA** (pmol g^-1^ FW)	**0.01**	**±**	**0.00**	**0.33**	**±**	**0.13**	***b***	**0.16**	**±**	**0.06**	***b***	*a*
**%**	**100%**			**3300%**				**1600%**				
**IAA** (pmol g^-1^ FW)	**2.07**	**±**	**1.13**	**3.75**	**±**	**0.95**		**2.19**	**±**	**1.65**		
**%**	**100%**			**181%**				**106%**				
CKs (pmol g^-1^ FW)												
Bases	13.70	±	3.54	15.54	±	4.16		7.79	±	3.09		
Ribosides	2.37	±	0.42	4.31	±	1.03	*a*	2.69	±	0.80		
Nucleotides	0.30	±	0.03	9.74	±	1.07	*c*	5.75	±	2.63	*a*	*a*
O-glucosides	118.22	±	20.92	301.23	±	37.84	*c*	243.84	±	57.27	*a*	
N-glucosides	7.05	±	1.20	33.03	±	8.59	*b*	20.10	±	9.58		
**Total CKs**	**141.22**	**±**	**20.86**	**363.85**	**±**	**39.68**	***c***	**280.16**	**±**	**73.17**	***a***	
	**100%**			**258%**				**199%**				
**Total BRs** (ng g^-1^ FW)	**17.80**	**±**	**2.37**	**38.14**	**±**	**3.46**	***b***	**27.88**	**±**	**2.10**	***a***	*b*
**%**	**100%**			**214%**				**157%**				
GAs (ng g^-1^ FW)												
13-Non-hydroxylated	25.85	±	5.34	35.12	±	13.10		40.41	±	11.85		
13-Hydroxylated	3.55	±	0.43	3.68	±	0.56		4.90	±	1.26		
Bioactive GAs	2.58	±	0.26	2.61	±	0.55		2.94	±	0.61		
**Total GAs**	**29.41**	**±**	**5.15**	**38.80**	**±**	**13.17**		**45.32**	**±**	**12.60**		
**%**	**100%**			**132%**				**154%**				

ABA, abscisic acid; IAA, indole-3-acetic acid; CKs, cytokinins; GAs, gibberellins; BRs, brassinosteroids.

Values are means of four (ABA, IAA, CKs) or three (GAs, BRs) biological replicates ± SD. Statistically significant differences determined using the two‐tailed Student’s t‐test are indicated with a, b or c, corresponding to P-values of 0.05 > P > 0.01, 0.01 > P > 0.001, and P < 0.001, respectively. Black letters indicate significant differences between watered controls and drought controls. Red letters indicate significant differences between drought controls and drought + MTU treatment. Bold text show percentage change in the value.

### Gene expression analysis

This description of the experiment is related to the results in **Table 3**. The same plant samples which were used for hormone analysis were used for gene expression analysis. Additionally, samples spray-treated by 10 μM TDZ were analyzed. Total RNA for reverse transcription was isolated using TRI Reagent^®^ Solution (Thermo Fisher Scientific Inc./Life Technologies, Waltham, MA, USA) and dissolved in 100 µL of nuclease-free water (Qiagen, Valencia, CA, USA). Isolated RNA was treated with 2 µL of TURBO™ DNase (Thermo Fisher Scientific Inc./Life Technologies, Waltham, MA, USA) for 30 min at 37°C followed by the addition of 1 µL of the enzyme for another 30 min. DNase-treated RNA was purified using Agencourt^®^ RNAClean^®^ XP to remove the enzyme. First-strand cDNA was synthesized using RevertAid™ H Minus Reverse Transcriptase (Thermo Fisher Scientific Inc./Life Technologies, Waltham, MA, USA) and an oligo(dT)_18_ primer (Sigma-Aldrich, St. Louis, MO, USA). After the transcription, cDNA samples were diluted in PCR grade water (Water, PCR Grade; Sigma-Aldrich, St. Louis, MO, USA) to 200 ng µl^-1^. Diluted cDNA samples were used as templates in real-time PCRs with SYBR™ Green chemistry. The reaction mixtures were prepared in a total volume of 5 µl that consisted of 2.5 µl SYBR^®^ Select Master Mix (Applied Biosystems, Foster City, CA, USA), 1.25 µl of forward and reverse primer mix (200 nM final concentration of each) and 1.25 µl of template cDNA (50 ng per reaction). cDNA samples (at least eight biological replicates) were run on a ViiA7™ Real-Time PCR System using a default program (Applied Biosystems) that included the following steps: 1. UDG activation phase (50°C for 2 min); 2. polymerase activation phase (95°C for 2 min); followed by forty cycles of 3. denaturation phase (95°C for 15 sec) and 4. annealing/extension phase (60°C for 1 min). The specificity of amplification was verified by melting curve analysis. The *TaGAPDH* gene (GenBank accession no. EU022331.1) and the *HvEF2* gene (homologous to *Triticum aestivum* elongation factor 1-beta, *TaEF1-b*; GenBank accession no. D13147.1) were used as endogenous controls to normalize variations in RNA quality and efficiency of transcription among the samples. Relative expression levels of the genes studied were analyzed according to Pfaffl (2001). References and the sequences of the primers used are given in **Supplemental Table S4**.

**Table 3:** Relative levels of selected gene transcripts in wheat shoots of 25-day-old plants after 5 days of drought treatment (SRWC was 72%).

Gene	Drought control		Drought + MTU		Drought + TDZ			
*TaCKX3*	0.8 ± 0.4		0.7 ± 0.3		0.7 ± 0.3			
***TaCKX4***	**2.3 ± 1.0**	*a*	1.9 ± 0.8		1.6 ± 1.1			
***TaGLU1***	**4.3± 1.9**	*c*	**4.8 ± 2.1**	*c*	**7.8 ± 3.3**	*b*		
***TaRR1***	0.8 ± 0.3		0.8 ± 0.5		**9.5 ± 2.7**	*a*	*b*	*b*
***TaRR4***	**0.5 ± 0.1**	*b*	**0.5 ± 0.2**	*b*	1.2 ± 0.6		*b*	*a*
***TaRR9***	**0.5 ± 0.2**	*b*	**0.5 ± 0.3**	*a*	**2.3 ± 0.6**	*b*	*c*	*b*
*TaCAT*	1.0 ± 0.7		0.8 ± 0.2		0.8 ± 0.2			
*TaSOD*	2.0 ± 0.3		1.9 ± 0.4		1.8 ± 0.3			
*TaSAG3*	2.0 ± 0.6		1.7 ± 0.3		1.1 ± 0.6			
*TaSAG5*	0.6 ± 0.5		0.8 ± 0.6		0.7 ± 0.6			
*TaSAG8*	0.9 ± 0.1		0.9 ± 0.2		2.4 ± 2.0			

Transcript accumulation was normalized against the levels of the TaGADPH and HvEF2 transcripts. Values are means of six biological replicates ± SD. Red and blue colors indicate up and down-regulation respectively, vs the watered control, whose transcript levels were defined to be 1. Bold lettering and numbers in boxes indicate statistically significant differences based on the two‐tailed Student’s t‐test; a, b, and c correspond to P-values of 0.05 > P > 0.01, 0.01 > P > 0.001, and P < 0.001, respectively. Black, red, and blue letters indicate significant differences between the watered control and the drought control, the drought control and drought + MTU treatments, and the drought + MTU and drought + TDZ treatments, respectively.

### Field experiments

**Figure 5:**
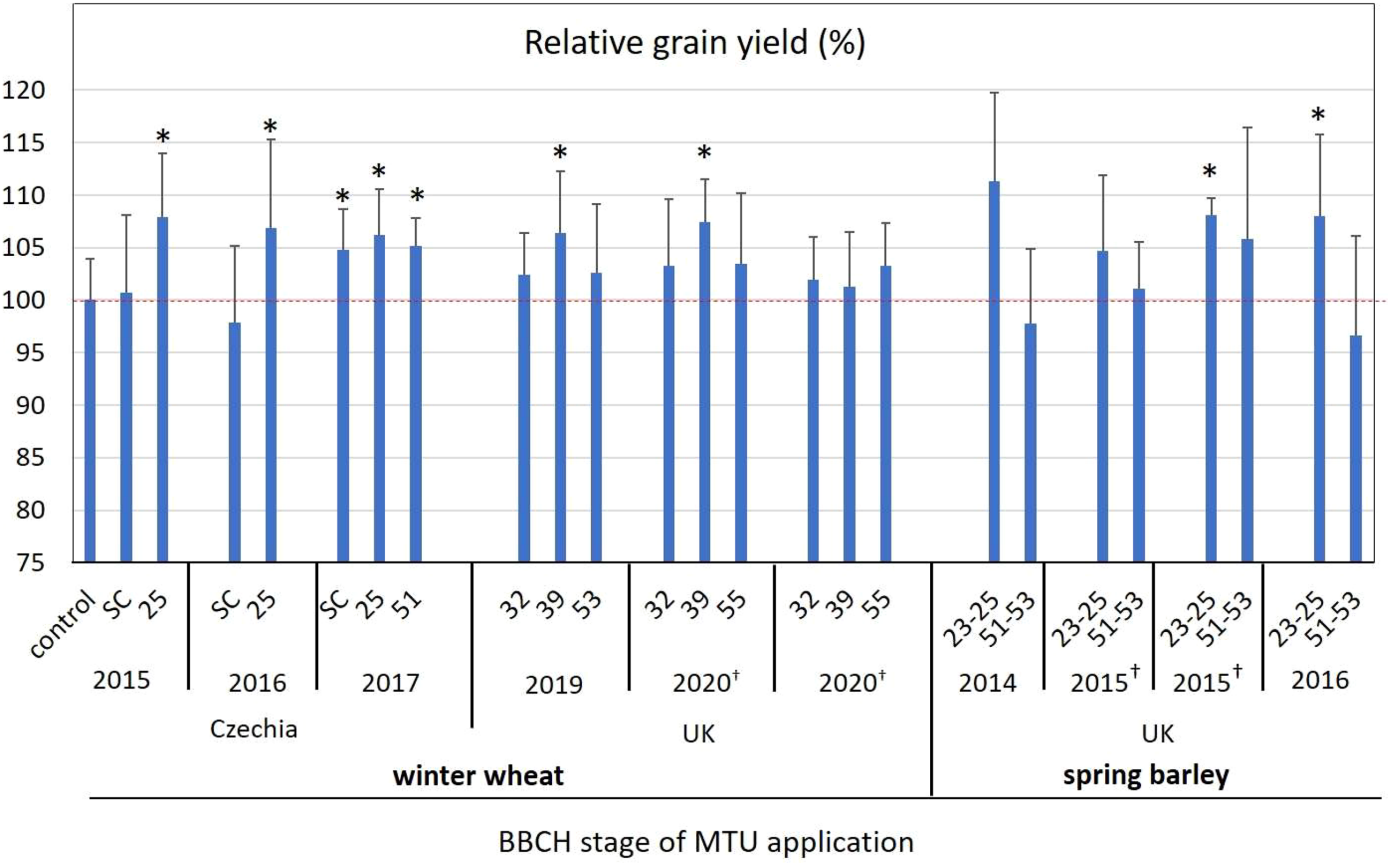
The effect of MTU on relative grain yield in field-grown winter wheat and spring barley. Values are means of five or six biological replicates ± SD. Asterisks indicate statistically significant differences determined by the two‐tailed Student’s *t*‐test at *P*-values of 0.15 > *P*. Red dotted line indicate 100% yield of untreated control. Complete data showing yields in t ha^-1^, values for all control treatments and thousand grain weight are presented in **Supplementary Table S5**. SC, seed coating; ^†^experiment was done at two different locations in the same region; UK, United Kingdom.

This description of the experiment is related to the results in **Figure 5** and **Supplementary Table S5**. All field experiments were done in accordance with the Good Experimental Practice criteria. In general, in experiments performed in the Czechia, MTU was applied by foliar spraying at a concentration of 5 μM and a dosage of 300 L ha^-1^ (0.3 g ha^-1^ of MTU). The concentration of MTU in the seed-coating mixture was 50 μM, and the mixture was applied at a dosage of 20 L per ton of seed. Winter wheat (cv. Etana and cv. Turandot) was used in 2015, and 2016 - 2017, respectively. Spring barley, cv. Bojos and cv. Francin was used in 2014, and in 2015 - 2016, respectively. All the trials were carried out in the Olomouc region, where wheat and barley production has a long history.

In experiments performed in the UK, MTU was applied by spraying at a concentration of 10 μM and a dosage of 200 L ha^-1^ (0.4 g ha^-1^ of MTU), which reflects the practices recommended for MTU-containing product in the UK. Winter wheat, cv. Barrel, Siskin, and Skyscraper were used in 2019, and 2020, respectively. The trials were carried out in North Yorkshire with the cultivars typical for the area and commercially available to the farmers in that year. Field trials conducted in the UK were performed and evaluated by the Eurofins company.

All field trials in Czechia and UK were performed in the same way. Six randomized plots of 10 m^2^ were used for the evaluation of one variant. Plots were laid out in the middle of the fields to avoid the edge effects. Except for MTU, no additive chemicals were used to treat the crops. Treatment timings are shown in **Figure 5** and **Table S5**.

### Additional notes

We are aware that both winter wheat and spring wheat and several different varieties (cultivars) were used during the work. This happened because the research was carried out over a period of 12 years, in two different states and in three different laboratories. The seeds of wheat were usually from local commercial sources and the sale of some varieties was discontinued during the research. However, increased greenness and delayed senescence were observed in all these varieties after MTU treatment, demonstrating its uniform and robust effect.

## Results

### Effects of MTU on wheat growth under optimal conditions

To assess the effect of MTU on chlorophyll content and growth of root and shoot in the medium term, winter wheat plants were grown in a greenhouse for 20 days and then sprayed with MTU. For comparison of MTU with cytokinin action, a part of the plants was sprayed with *trans-*zeatin (*t*Z). After the treatment, the relative chlorophyll content was monitored regularly using a SPAD meter (**Figure 1A**) until the plants were harvested and their weight parameters were determined (**Table 1**). Both compounds increased chlorophyll contents by 6-10% during the first 10 days after application and by 11-18% on days 15-25 after application. These data represent the chlorophyll content in a fixed area of a leaf. We therefore also computed the chlorophyll coefficient per plant, which is defined as the product of the shoot dry weight (g) and the SPAD value. Both treatments significantly, and to the same extent increased chlorophyll coefficient as well as the root and shoot dry weight when compared to the controls. Their effect on root growth was more pronounced than the effect on shoot growth (**Table 1A** and **Supplemental Figure S1**). In a second similar experiment, MTU also improved all studied parameters, but its effect on root growth was weaker than in the first one (compare the results in **Tables 1A, B**).

### Effects of MTU on wheat growth under heat and drought stress

Under heat stress, *t*Z and MTU strongly and equally retarded degradation of chlorophyll, causing the treated plants’ relative chlorophyll contents to be approximately 20, 30, and 40% higher than those in untreated controls on days 15, 20, and 25 during stress, respectively (**Figure 1B**). However, root and shoot dry weights were higher after MTU treatment than after *t*Z treatment. Interestingly, the roots of MTU-treated plants were 37% heavier than the roots of stressed control plants (**Table 1A**). The chlorophyll coefficient was slightly higher in MTU-treated plants, mainly due to the fact that MTU also improved shoot growth but *t*Z did not. This effect also contributed to a higher total dry weight of MTU-treated plants compared to *t*Z-treated plants. Representative examples of root systems of control and MTU-treated plants are shown in **Supplemental Figure S2**.

Under mild drought stress (plants were watered gently when wilting, soil relative water content (SRWC) was 70 – 60%), chlorophyll content in stressed control plants declined during 25 days by 8% when compared to watered control plants. MTU completely reverted this effect and kept the chlorophyll content in stressed plants at the same level as in watered control plants (**Figure 1C**). Remarkably, MTU increased the root dry weight by 42%, so the roots had the same weight as in MTU-treated unstressed plants. As before, shoot dry weight and chlorophyll coefficient were also improved (**Table 1B**). In another experiment, where 14-day-old wheat plants were exposed to drought stress for 7 days (SRWC decreased to 23%), MTU-treated plants contained more than twice as much chlorophyll (209%) as untreated plants (**Figure 1D**), and after re-watering recovered faster (**Figure 1E**) and had a higher survival rate (**Figure 1F**).

### MTU inhibited age-related and drought-induced senescence

To better understand the mechanism of improving the growth and stress tolerance of MTU-treated wheat, we analyzed pigment contents and photosynthetic parameters in the first leaves of wheat seedlings. MTU was applied to the nutrient solution where seeds germinated, so its effect on the early development of the first leaf could be observed. The chlorophyll content in the first leaves of control/MTU-treated plants peaked 7 days after planting; at this time point, leaves of MTU-treated plants contained 4% more chlorophyll than control plants. This difference grew until day 20 when MTU-treated plants contained 20% more chlorophyll (**Figure 2A**) and 13-22% more carotenoids (**Figure 2B**). From **Figure 2A**, it is clear that chlorophyll in the first leaf had been declining since the seventh day. This reduction is genetically controlled and is part of age-related programmed senescence (Woo et al., 2013). MTU clearly suppressed this process.

The analysis of photosynthetic parameters revealed that the high content of chlorophyll was accompanied by enhanced effective quantum yields of PSI [Y(I)] and PSII [Y(II)] photochemistry in the light-adapted state (**Figures 3A–C** and **Supplemental Figures S3**, **S4**). Both parameters Y(I) and Y(II) were lower in 21-day-old leaves than in 14-day-old leaves and drought stress caused their further decrease in all treated variants. MTU treatment significantly alleviated this decrease. Moreover, the difference in the Y(I) and Y(II) between MTU-treated and control plants was more significant in drought-stressed plants (compare the parameters in 21-day-old leaves in the absence and presence of drought stress). Notably, Y(II) was 17% higher in MTU-treated plants than in control plants on day 14.

A striking difference was observed in the cyclic electron flow (CEF) around the PSI (**Figure 3D** and **Supplemental Figure S5**). After MTU treatment CEF was 50% higher in the 21-day-old leaves in the absence of stress and almost doubled in water-deficit plants (+ 84%). We can conclude that MTU markedly enhanced the CEF and the activity of photosystems in drought-stressed plants. In addition, MTU treatment lowered non-photochemical quenching, and the 1-qP parameter, which reflects a fraction of the reduced PQ pool in thylakoid membranes when compared to the control treatment (**Figures 3E, F** and **Supplemental Figures S6**, **S7**). This indicates lower excitation pressure on PSII and higher electron flow from PSII in the MTU-treated leaves. Consistent with the lower limitation of photosynthetic activity, the shoot dry weight of stressed plants treated with MTU was higher (+30%) than that of control plants, which is consistent with the results obtained after foliar application (**Table 1B**). A decrease in leaf water potential was similar in all stressed plants (**Supplemental Figure S8**), indicating that MTU did not reduce plant water deficit under drought.

In contrast to the effect of MTU, treatment with *t*Z showed no positive effect on any of the analyzed photosynthetic parameters (**Figure 3** and **Supplemental Figures S3**-**7**). However, a control wheat leaf senescence assay with the same *t*Z solution confirmed its activity (**Supplemental Figure S9**), so it seems that *t*Z has a protective effect when applied to leaves, but not when applied to roots.

### MTU increased the abundance of PSI-LHCa supercomplexes

To clarify the observed protective effect of MTU on the function of photosynthetic apparatus, the composition of the chlorophyll-binding membrane proteins in 21-day-old wheat plants was analyzed by 2D clear-native/SDS electrophoresis. Interestingly, when samples were loaded so as to equalize their chlorophyll content on a dry weight basis, we observed a higher abundance of PSI-LHCa supercomplexes in MTU-treated plants than in controls (**Figure 4**). However, both groups had similar contents of light-harvesting complexes II (LHCb), which typically bind the majority of the chlorophyll molecules in the chloroplast (Bowsher et al., 2008). Additionally, the content of PSII in treated plants was not greatly increased, although there was a visible difference in the contents of large PSII-LHCb supercomplexes (**Figure 4**).

We concluded that treatment with MTU enhanced all chlorophyll-binding protein complexes to some extent, but the content of PSI-LHCa supercomplexes, in particular, has increased substantially relative to that in control plants.

### Analysis of hormone content in wheat plants after MTU treatment

To examine the possible involvement of plant hormones in MTU action, the levels of abscisic acid (ABA), cytokinins, auxin - indole-3-acetic acid, brassinosteroids (BRs), and gibberellins (GAs) were determined in watered and drought-stressed control and drought-stressed and MTU-treated plants.

A positive upward trend was seen for all groups of plant hormones analyzed in stressed samples (**Table 2**). The increase (except for that for GAs) was smaller in plants treated by MTU. Thus, stressed MTU-treated plants contained twice as less ABA than stressed control plants. This reflects the reduced stress response in MTU-treated plants because ABA is a major phytohormone signal produced in response to drought stress (Cutler et al., 2010). In comparison with watered control plants, the content of indole-3-acetic acid was 81% higher in stressed control, while only 6% higher in stressed and MTU-treated plants. Similarly, the content of total cytokinins was 160% higher in stressed control, but only 100% higher in stressed and MTU-treated plants. In all cases, the majority of total cytokinins were present as inactive *O*-glucosides, whose levels increased significantly in all stressed samples (for details see **Table 2** and **Supplemental Table S1**). In conclusion, stressed MTU-treated plants had lower levels of cytokinins than stressed control plants but showed less evidence of senescence. Therefore, cytokinins could not be responsible for the effect of MTU. More likely, it seems that the changes in endogenous cytokinin levels following MTU treatment merely reflected the plants’ physiological state.

The total levels of the fifteen natural BRs analyzed in this work showed the same trends as those seen for ABA and cytokinins: levels of endogenous BRs were lowest in watered controls, somewhat higher (157%) in stressed MTU-treated plants, and roughly two-fold higher (214%) in stressed controls (**Table 2**, for detail, see **Supplemental Table S2**). BRs are known to enhance plants’ tolerance to various abiotic stresses by increasing the levels of various ROS scavenging components (reviewed by Vardhini and Anjum, 2015). Therefore, and as well as in the case of cytokinins, the lower content of BRs in stressed and MTU-treated plants (compared to stressed controls) could not be responsible for the higher drought tolerance of MTU-treated plants.

GAs are also involved in plant stress responses; several authors have found that suppression of GA signaling is a general response to abiotic stress, and plants’ sensitivity to water and salinity stress increases as GA levels increase (for a review, see Colebrook et al., 2014). We analyzed the levels of eight 13-non-hydroxylated GAs and ten 13-hydroxylated GAs (**Table 2**; for details, see **Supplemental Table S3**). As with other hormone levels, the total level of GAs was also increased in all drought-stressed samples, with that the MTU-treated plants contained a bit more GAs than stressed control plants. The levels of bioactive GAs (GA_1,_ GA_3_, GA_4_, GA_5_, GA_6_, and GA_7,_ Yamaguchi, 2008) were almost the same in all treatments, including watered controls. Our results generally indicate that drought stress enhances the production of GAs in wheat, making it more sensitive to stress. However, MTU treatment reduces plant sensitivity to stress, strongly suggesting that the senescence-delaying effect of MTU prevails over the senescence-promoting effect of GAs.

**Figure 6:**
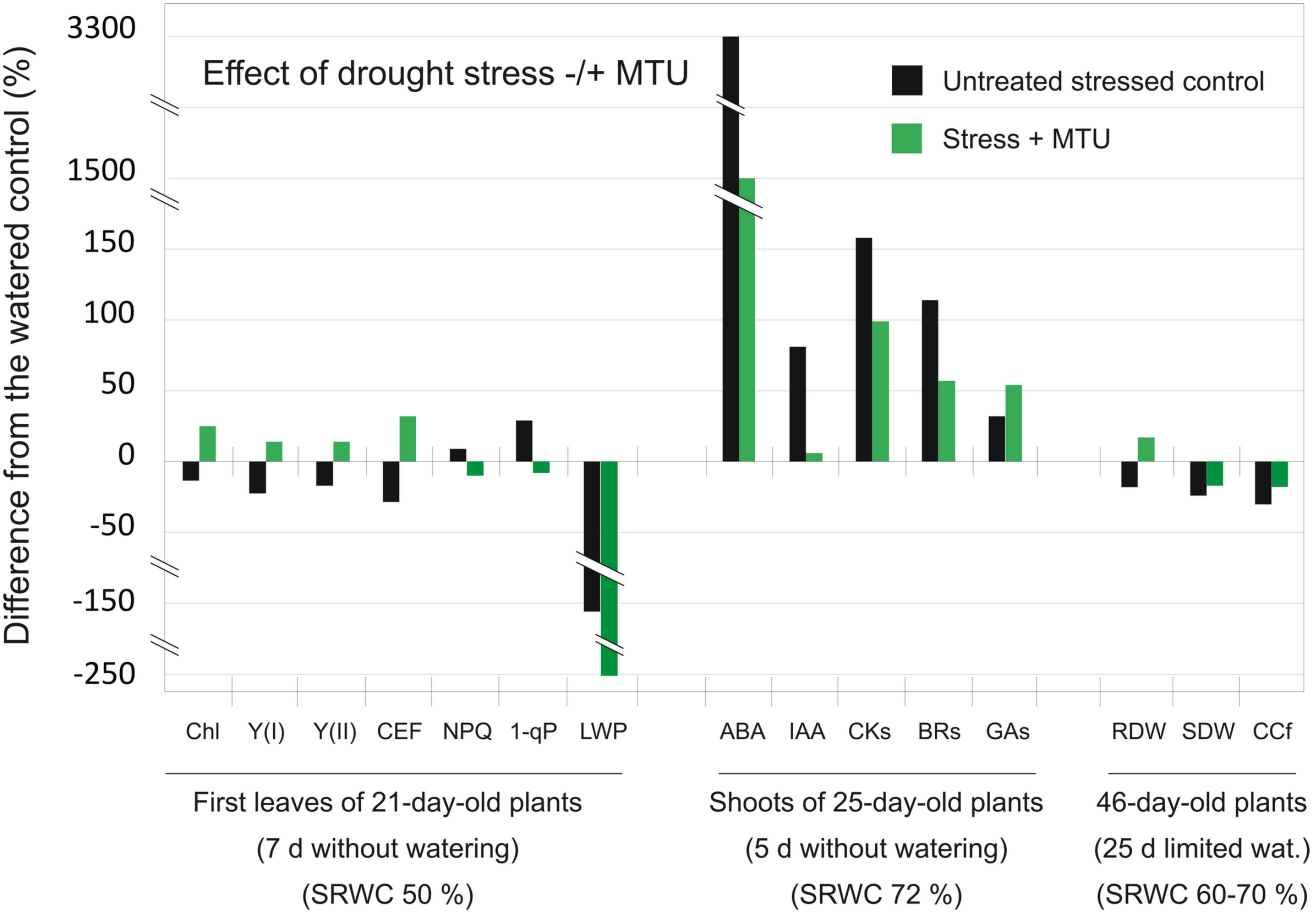
The comparison of relative differences in individual parameters and phytohormone content induced by drought in control plants and MTU-treated plants relative to the watered control plants. Chl – chlorophyll content; Y(I) and Y(II) – the effective quantum yield of PSI and PSII photochemistry in the light-adapted state; CEF, cyclic electron flow around PSI; NPQ, non-photochemical quenching; 1-qP, a fraction of reduced PQ pool; LWP – leaf water potential; ABA, abscisic acid; IAA, indole-3-acetic acid; CKs, cytokinins; BRs, brassinosteroids; Gas, gibberellins; RDW, root dry weight; SDW, shoot dry weight; CCf, chlorophyll coefficient (defined as the product of the shoot dry weight (g) and the SPAD value); d, days; wat., watering; SRWC, soil relative water content.

Taken together, our results suggest that levels of all tested phytohormones are elevated in wheat plants subject to water deficiency and that MTU treatment reduced this increase (except those for GAs) (**Figure 6**). Therefore, if hormone levels rise in response to stress, the results clearly show that MTU-treated plants suffer less than control plants so that MTU increases their drought tolerance.

The overall effect of drought stress, without or with simultaneous MTU treatment, on individual photosynthetic and growth parameters, and phytohormone content is clearly summarized in **Figure 6**. This Figure also summarizes the experimental design used in this work.

### Analysis of gene expression in wheat plants after MTU treatment

To strengthen the conclusions of the hormone level analysis and distinguish between the effects of MTU and cytokinins, the expression of selected genes was investigated in plants exposed to drought stress and treated with either MTU or TDZ. TDZ was chosen because it has a stronger protective effect than *t*Z (Nisler et al., 2018) and shares the same structural motif with MTU. The chosen genes encode products associated with cytokinin metabolism (TaIPT5, TaIPT8, TaCKX3, TaCKX4, TaCKX6, TaGLU1, TaGLU2) and response (TaRR1, TaRR4, TaRR9), antioxidant defense (TaCAT, TaSOD) and senescence (TaSAG3, TaSAG5, and TaSAG8). Transcripts of *TaIPT5*, *TaIPT8, TaCKX6*, and *TaGLU2* were not detected, while the transcripts of *TaCKX3*, *TaCAT*, *TaSOD*, *TaSAG3*, *TaSAG5*, and *TaSAG8* exhibited no significant differences between samples. However, *TaGLU1* (glucosidase 1) expression was significantly increased in all plants subjected to drought, and *TaCKX4* expression was elevated in drought-treated control plants. The greatest differences were observed for cytokinin primary response genes (*TaRRs*): the expression of *TaRR1* was roughly 12 times higher in TDZ-treated plants than in other treatment groups, and similar trends were observed for *TaRR9* and *TaRR4*. Stressed plants treated with MTU or TDZ thus exhibited delayed leaf senescence, whereas the expression of all studied *TaRR* transcripts was reduced in MTU-treated plants, it was increased in TDZ-treated plants (**Table 3**). TDZ thus apparently delays senescence *via* the activation of cytokinin primary response genes and subsequent regulation of cytokinin-dependent processes, whereas MTU acts *via* a different mechanism.

### The effect of MTU on grain yields of field-grown barley and wheat

Since the application of MTU had a significant positive effect on plant growth in laboratory and greenhouse conditions, the effect of MTU on growth and the yield of field-grown wheat and barley was investigated.

Wheat: During the 2015 – 2017 growing seasons in the Czech Republic, MTU treatment improved average grain yields by 1.1% and 7.0% when applied by seed coating, or at the tillering stage (BBCH 23-25), respectively (**Figure 5** and **Table S5**). In the United Kingdom, MTU treatment during the 2019 and 2020 growing seasons increased the average grain yield by 2.5%, 5.0%, and 3.1% when applied at the BBCH 32, BBCH 39, and BBCH 50-55 growth stages, respectively (**Figure 5** and **Table S5**).

Barley: During the three harvest years 2014 - 2016 in the Czechia, MTU increased the average yield of grains by 8.0%, and 1.3% when applied at the tillering stage (BBCH 23-25), or at the inflorescence emergence stage (BBCH 51-53), respectively (**Figure 5** and **Table S5**). In accordance with the data presented in **Table 1**, MTU-treated plants had significantly (at *P* < 0.01) higher dry weights by 52% at BBCH 30 than controls due to equal increases in shoot and root weight. Notably, plants treated with MTU at the tillering stage produced on average 8% more viable tillers and contained significantly lower cytokinin levels (**Supplemental Table S5**) than control plants.

In conclusion, MTU increased the grain yield of barley and wheat most effectively (by 5-8%) when applied at BBCH 25 and 39 (tillering and stem elongation stages) rather than when applied at Inflorescence emergence (BBCH 51-55). These results were achieved with treatment at 0.4 g ha^-1^ MTU (foliar application of 10 μM MTU at a dose of 200 L ha^-1^). At this dosage, MTU has also greening effects (**Table 1** and **Figures 2A**, **3A**), and improves stress tolerance (**Figures 1**–**3**). Additionally, MTU was found to be non-ecotoxic (to soil arthropods, invertebrates, and soil microbial community) in an evaluation conducted by the Czech Central Institute for Supervising and Testing in Agriculture (**Data S1**). Given that MTU has a simple molecular structure, contains no halogen atoms, and is effective at very low doses, it has become a commercially available eco-friendly biostimulant in the UK in 2022. In 2023 MTU will be available also in Czechia, Poland, and Hungary and in the future years in other EU countries.

## Discussion

Based on the current knowledge and our current results, we propose that during age-related as well as during stress-induced senescence MTU primarily blocks the process that leads to PSI-LHCa disintegration (**Figure 4**). And, it seems that PSI disintegration in cereals occurs at the very beginning of the senescence process (Miersch et al., 2000; Tang et al., 2005). Our data support this claim when showing here that MTU specifically increased the content of PSI-LHCa in young wheat plants and simultaneously postponed the degradation of dimeric PSII and PSII-LHCII supercomplexes in leaves kept in darkness (Nisler et al., 2018). Supercomplexes of PSI were, however, missing in all samples, indicating a protective role of PSI on PSII in wheat.

What we found at the protein level (**Figure 4**) is consistent with what we observed at the functional level, i.e. enhanced CEF (**Figure 3D**), higher efficiency of both photosystems (**Figures 3B, C**) and lower NPQ (**Figure 3E**). We believe that this is mainly the effect of the higher CEF, which by a feedback loop prevents (photo)oxidative damage of other chloroplast components. As noted by Zivcak et al. (2014), CEF around PSI plays a key role in reducing the risk of oxidative stress in plants exposed to drought stress. The genetic evidence suggests that ROS, besides causing physicochemical damage, act as a signal that triggers senescence (Foyer and Noctor, 2005) also by an elevation of free ABA levels (Huang et al., 2012; Boursiac et al., 2013; Song et al., 2014). Consistently, we found a significantly lower concentration of ABA in MTU-treated leaves exposed to drought stress than in untreated leaves. Also, we have determined lower oxidative damage and lower ethylene production in the senescent leaves treated with MTU in comparison to control leaves or the leaves treated with TDZ (Nisler et al., 2018). It is thus highly probable that MTU negatively regulates the onset of senescence by decreasing the concentration of ROS in leaves.

Considering that MTU significantly delayed the senescence induced by various stimuli including wounding, darkness, salinity (Nisler et al., 2018), drought, heat, and age, this work indicates a central role of PSI and CEF in the onset and/or progress of senescence. Some other reports suggested that improvement in CEF led to higher drought (Long et al., 2008; Lehtimaki et al., 2010) and salt stress tolerance (He et al., 2015). In soybean (*Glycine max*) the enhanced CEF improved salt tolerance by supplying extra ATP that was utilized to sequester Na^+^ in the vacuole instead of the chloroplast (He et al., 2015). MTU thus may have played the same role in wheat, which exhibited significantly delayed leaf yellowing after MTU application (Nisler et al., 2018). CEF seems to also play an important role in the early developmental stages, however, its role during leaf development and senescence is still poorly understood and can differ among species (Suorsa, 2015; Krieger-Liszkay et al., 2019). Generally, it has been suggested that CEF increases in response to various stresses (Golding and Johnson, 2003; Golding et al., 2004) as well as the content of components responsible for CEF operation (Lehtimaki et al., 2010; reviewed by Suorsa, 2015). However, as Johnson (2011) noted, there is no clear evidence, that increases in *PGR5* (PROTON GRADIENT REGULATION5 - one of the components mediating CEF) expression or PGR5 protein will actually give rise to increased CEF, and that overexpression would suggest the opposite. Here we report a decline of CEF in senescing/drought-stressed wheat (**Figure 3D**) which is not in agreement with the above-cited literature. However, consistently with others, we show that improvement in CEF (by MTU) provides enhanced drought tolerance (**Figure 3**). Moreover, we show for the first time that enhanced CEF can delay the programmed age-induced senescence in wheat (**Figures 2**, **3**), which allows plants to grow faster (**Table 1**) and produce a higher yield (**Figure 5**). This work thus significantly expands our insight into the role of PSI/CEF in plant development and yield management.

Given that the highest anti-senescence (anti-stress) activity was always attributed to cytokinins (Gan and Amasino, 1995; Gan, 2003) and that MTU exhibited low to zero cytokinin activity in wheat and Arabidopsis (Nisler et al., 2018), the observed effect of MTU is remarkable. In this work, we provide additional clear evidence that MTU does not utilize the cytokinin signaling pathway. Likewise, other groups of hormones do not appear to be directly involved. Since the positive effect of MTU is associated with a function of PSI it is very unlikely that it involves a cytokinin mode of action, as there is no evidence in the literature that cytokinins have a positive effect on the stability or activity of PSI. On the contrary, there is a plethora of evidence on genetics, protein, and functional levels that cytokinins protect PSII (e.g. Cortleven et al., 2014; Talla et al., 2016; Janečková et al., 2019). Thus, MTU is the only chemical reported up to date, to enhance the abundance and activity of PSI.

How MTU stimulates the accumulation of PSI-LHCa supercomplexes is unclear. The level of PSI in plants is generally very stable (Schöttler and Tóth, 2014), a higher level of PSI has only been reported after acclimation to very low light conditions or long-term exposure to light exciting specifically PSII complexes (Dietzel et al., 2011). These two exceptions aside, plants adjust photosystem stoichiometry by altering the levels of PSII (Schöttler and Tóth, 2014). To our knowledge, there is only one mutant plant line - an Arabidopsis MORC2 (Microrchidia-type ATPase) mutant - that has elevated levels of both PSI and LHCII antennae without any concomitant increase in PSII levels (van Tol et al., 2017). Also, there are no reported chemical agents that cause specific accumulation of PSI-LHCa complexes in plants. Therefore, MTU is a unique chemical tool for future studies of PSI, CEF, and their involvement in senescence regulation in various crop species. How MTU operates on the molecular level needs to be addressed in future studies.

Regarding the agronomic potential of MTU, it is obvious that MTU, by prolonging the photosynthetically active lifetime of the leaves (**Figures 1**, **3**), increases carbon assimilation which is reflected in higher dry matter production and yield (**Table 1** and **Figure 5**). The close relationship between total plant net CO_2_ assimilation and dry weight yield was confirmed previously (Peterson and Zelitch, 1982). The finding that MTU worked most effectively when applied in the early growth stages and when plants are exposed to stress conditions is consistent with an observation that premature/earlier leaf senescence even in early developmental stages has a major influence on yield formation in wheat and maize (El Hafid et al., 1998; Li et al., 2019). Lafarge et al. (2002) further showed that higher levels of photo-assimilates favor the promotion of tiller bud growth, which agrees with our observation in field-grown barley. The observed effect of MTU on tillering and yield is therefore in line with claims that increased photosynthesis translates into greater crop yields (reviewed in Carmo-Silva et al., 2017). Based on the data presented here, we believe that MTU is a perfect biostimulant that can provide an eco-friendly solution to the global need for more efficient and sustainable agriculture.

## Data availability statement

The datasets presented in this study can be found in online repositories. The names of the repository/repositories and accession number(s) can be found in the article/**Supplementary Material**.

## Author contributions

JN conceived the project and wrote the article, with contributions from all the authors. RS and JP analyzed protein complexes and pigment content. ZK, MŠp, and AH measured and analyzed the photosynthetic parameters. RK conducted field trials in CZ. JS performed the gene expression analysis. MŠk conducted a statistical analysis of the results. SR provided the phenotype analysis of plants cultivated in optimal, and under heat and drought stress conditions. DT and ON determined the content of individual plant hormones. MS supervised the project. All authors contributed to the article and approved the submitted version.
