# Supplementary material for: Urea derivative MTU improves stress tolerance and yield in wheat by promoting cyclic electron flow around PSI
Source: Front Plant Sci. 2023 Mar 7;14:1131326. doi: 10.3389/fpls.2023.1131326 (PMC10028069; doi:10.3389/fpls.2023.1131326)
Supplement: Supplementary file 1 [file DataSheet_1.pdf]

Supplementary data

Article title: Urea derivative MTU improves stress tolerance and yield in wheat by promoting cyclic electron flow around PSI

*Jaroslav Nisler[1], Zuzana Kučerová[2], Radoslav Koprna[3], Roman Sobotka[4], Jana Slivková[5], Stephen Rossall[6], Martina Špundová[2], Alexandra Husičková[2], Jan Pilný[4], Danuše Tarkowská[7], Ondřej Novák[7], Mária Škrabišová[5], Miroslav Strnad[7]

The following Supplementary data are available for this article:

Figure S1. Representative root systems of wheat plants grown under optimal conditions.

Figure S2. Representative root systems of wheat plants grown under heat stress conditions.

Figure S3. Light-induced kinetics of effective quantum yield of PSI [Y(I)].

Figure S4. Light-induced kinetics of effective quantum yield of PSII [Y(II)].

Figure S5. Light-induced kinetics of cyclic electron flow around PSI (CEF).

Figure S6. Light-induced kinetics of non-photochemical quenching (NPQ).

Figure S7. Light-induced kinetics of 1-qP (the fraction of reduced PQ pool).

Figure S8. Shoot dry weight and leaf water potential of wheat plants.

Figure S9. The effect of *trans*-zeatin on chlorophyll retention in wheat leaf senescence assay.

Table S1. Endogenous levels of individual cytokinin forms in intact wheat leaves.

Table S2. Endogenous levels of brassinosteroids (ng g^{-1} FW) in intact wheat leaves.

Table S3. Endogenous levels of gibberellins (ng g^{-1} FW) in intact wheat leaves.

Table S4. List of primers used in this work and corresponding references.

Table S5. The effect of MTU on yield and yield-forming parameters in field-grown winter wheat and spring barley.

Table S6. Endogenous levels of individual cytokinin groups in intact barley leaves.

Data S1. Eco-toxicity test results for MTU.

Fig. S1. Representative root systems of wheat plants grown under optimal conditions. Control plants (left) and plants treated with 10 μM MTU (right).

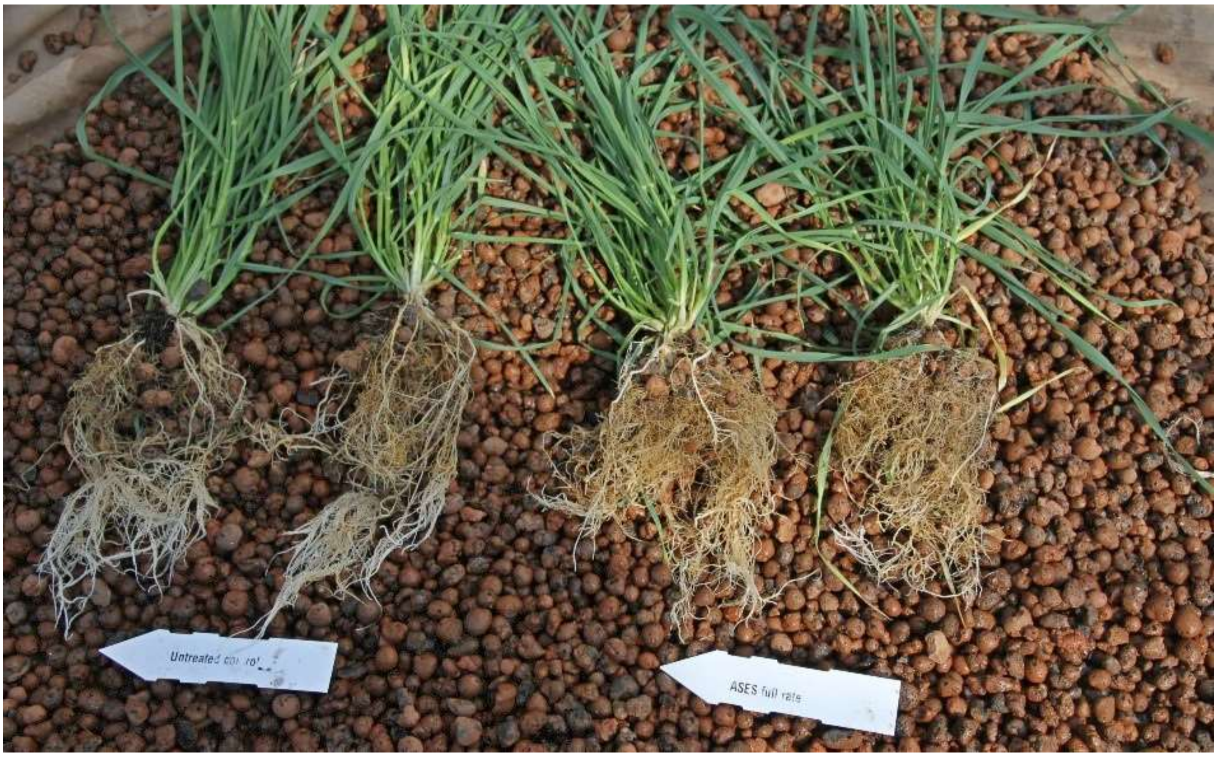

Fig. S2. Representative root systems of wheat plants grown under heat stress conditions. Control plants (left) and plants treated with 10 μM MTU (right).

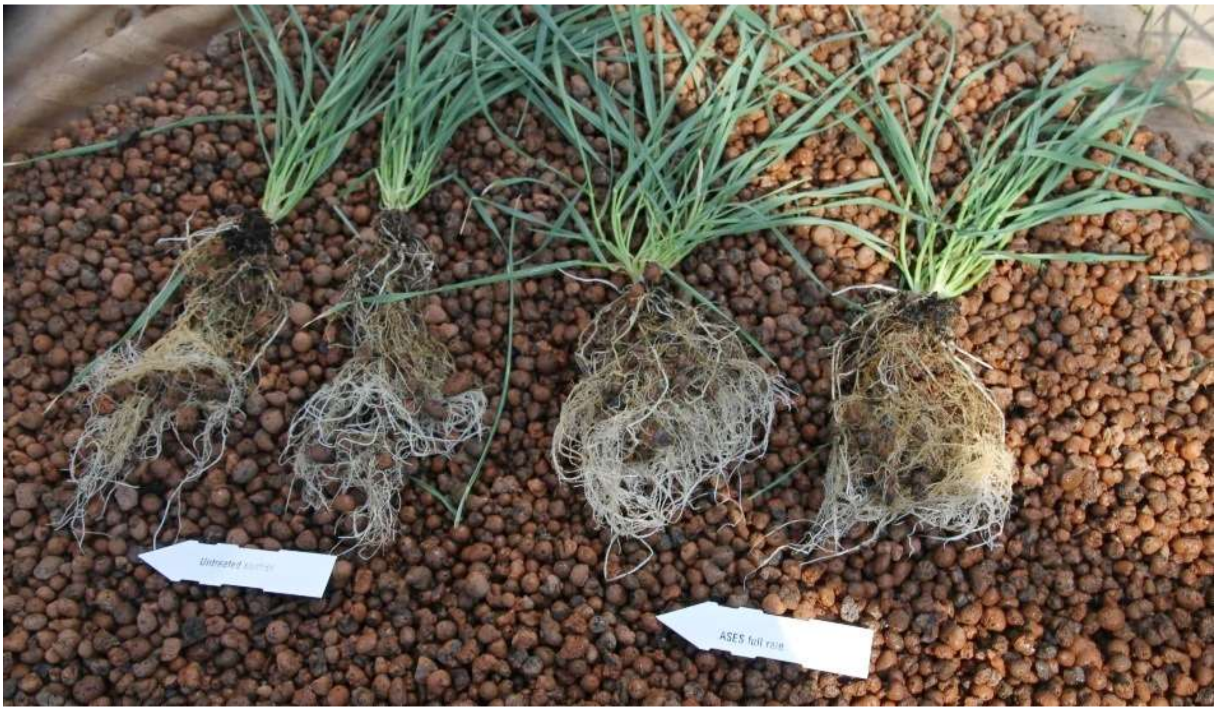

Fig. S3. Light-induced kinetics of effective quantum yield of PSI (YI) in the first leaves of 14-day-old and 21-day-old wheat plants grown under optimal conditions and in the first leaves of 21-day-old wheat plants grown in water deficit. Plants were treated with *trans*-zeatin (*t*Z) or MTU. Values are means ± SD of five biological replicates. The legend in the first figure applies to all figures.

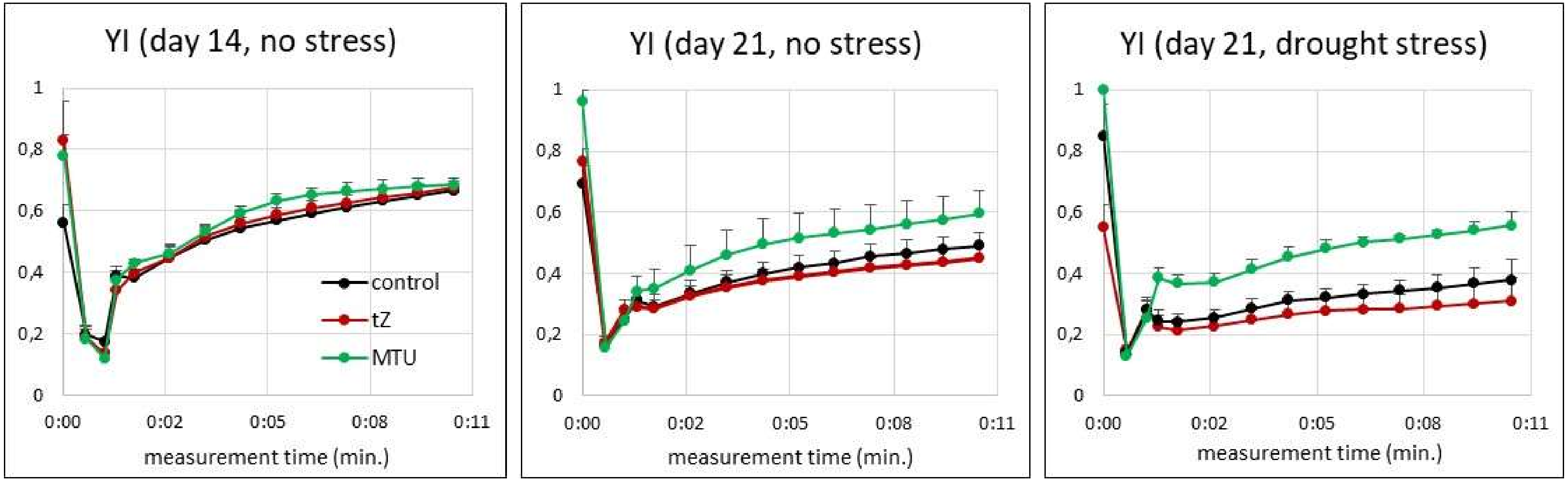

Fig. S4. Light-induced kinetics of effective quantum yield of PSII (YII) in the first leaves of 14-day-old and 21-day-old wheat plants grown under optimal conditions and in the first leaves of 21-day-old wheat plants grown in water deficit. Plants were treated with *trans*-zeatin (*t*Z) or MTU. Values are means ± SD of five biological replicates. The legend in the first figure applies to all figures.

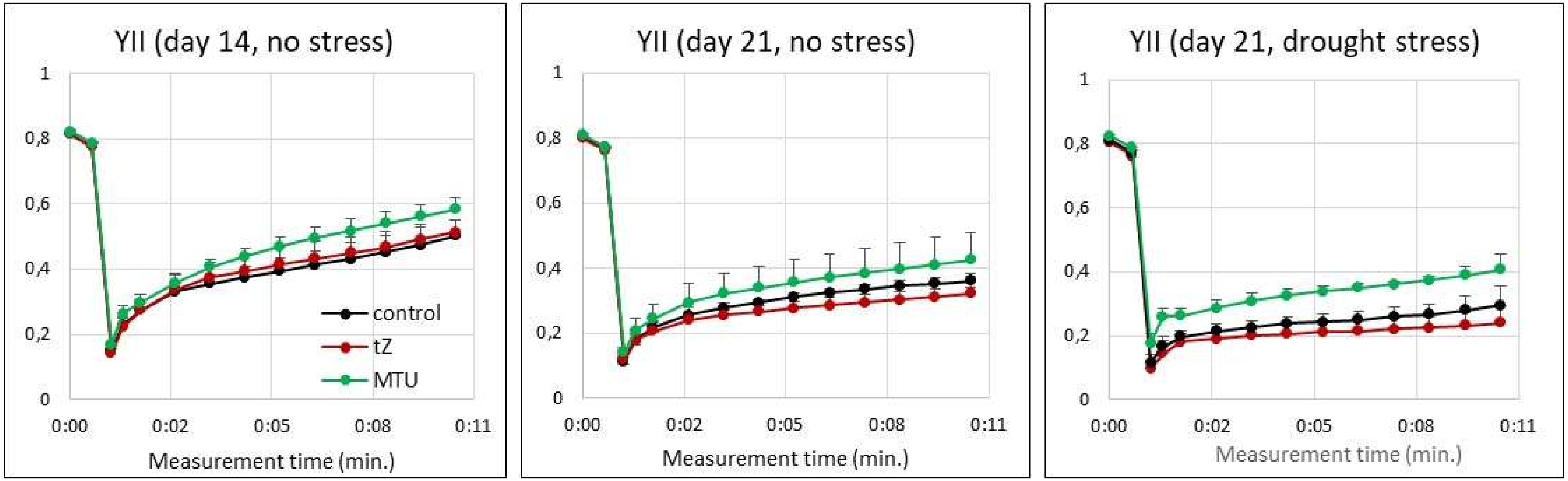

Fig. S5. Light-induced kinetics of cyclic electron flow around PSI (CEF) in the first leaves of 14-day-old and 21-day-old wheat plants grown under optimal conditions and in the first leaves of 21-day-old wheat plants grown in water deficit. Plants were treated with *trans*-zeatin (*t*Z) or MTU. Values are means ± SD of five biological replicates. The legend in the first figure applies to all figures.

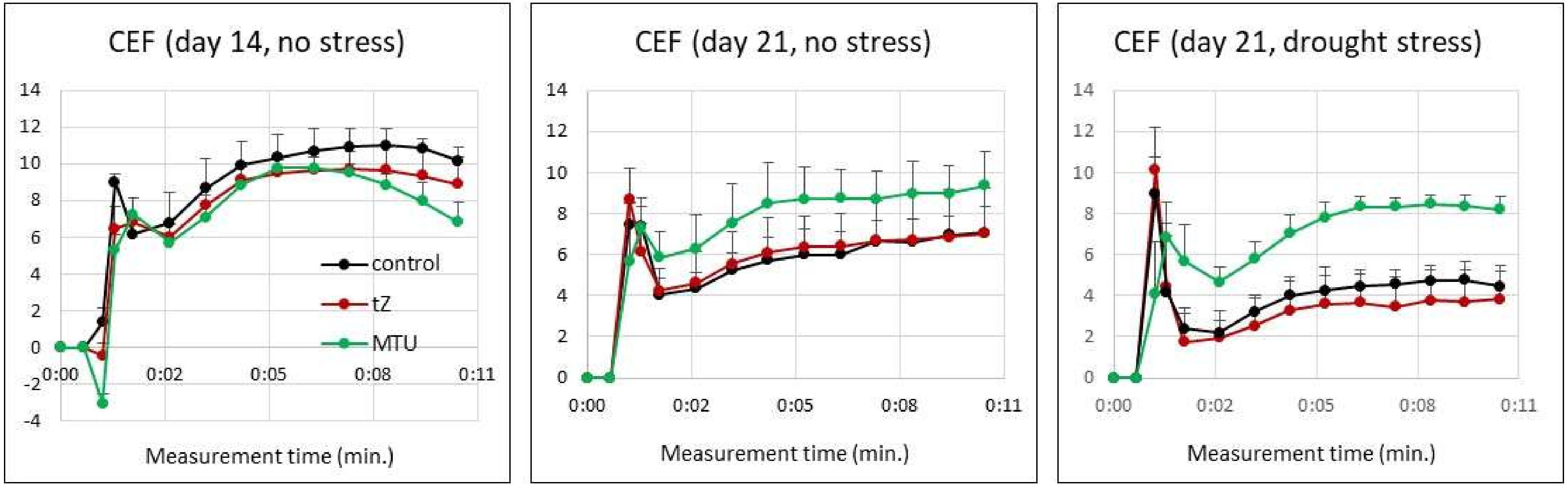

Fig. S6. Light-induced kinetics of Non-photochemical quenching (NPQ) in the first leaves of 14-day-old and 21-day-old wheat plants grown under optimal conditions and in the first leaves of 21-day-old wheat plants grown in water deficit. Plants were treated with *trans*-zeatin (*t*Z) or MTU. Values are means ± SD of five biological replicates. The legend in the first figure applies to all figures.

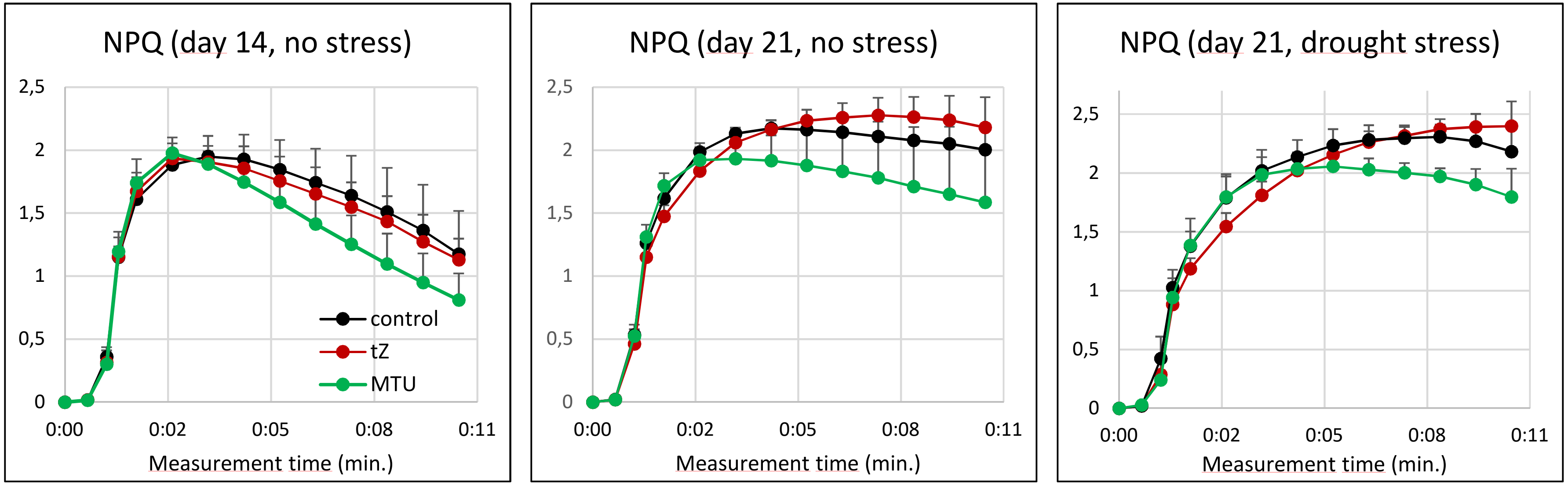

Fig. S7. Light-induced kinetics of 1-qP in the first leaves of 14-day-old and 21-day-old wheat plants grown under optimal conditions and in the first leaves of 21-day-old wheat plants grown in water deficit. Plants were treated with *trans*-zeatin (*t*Z) or MTU. Values are means ± SD of five biological replicates. The legend in the first figure applies to all figures.

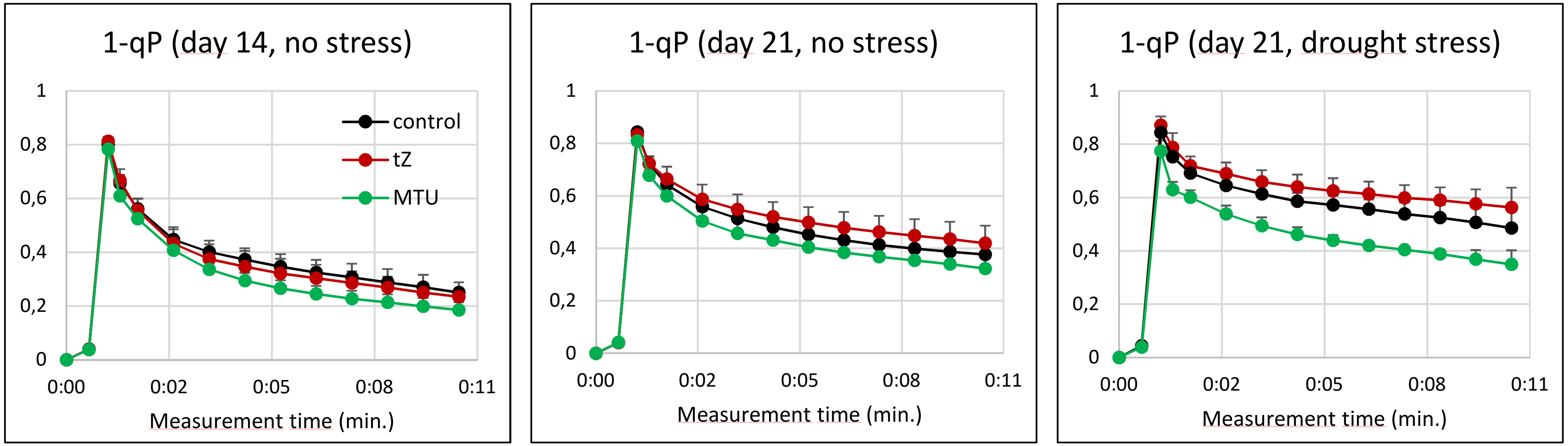

Fig. S8. Shoot dry weight (**A**) and water potential of the first leaves (**B**) of 21-day-old wheat plants treated with *trans*-zeatin or MTU under optimal and drought stress conditions. Values are means ± SD of ten (**A**) and five (**B**) biological replicates. Asterisk in A indicates a statistically significant difference between untreated control and treatment at $P < 0.01$ based on the two-tailed Student's *t*-test.

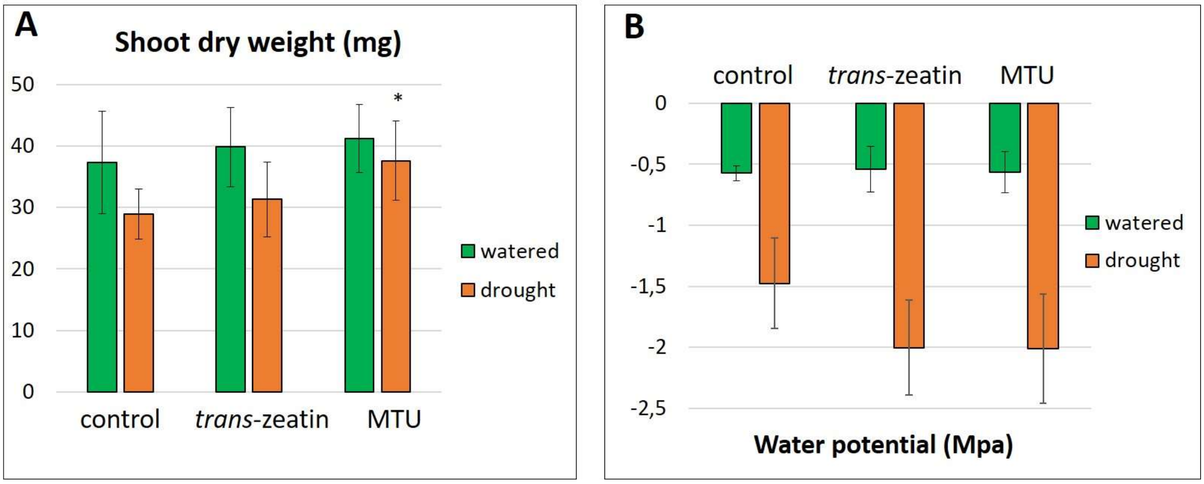

Fig. S9. The effect of *trans*-zeatin on chlorophyll retention in wheat leaf senescence assay. (**A**) The appearance of the wheat leaves after seven days in the dark. (**B**) Chlorophyll level in the leaves as determined by SPAD meter reading.

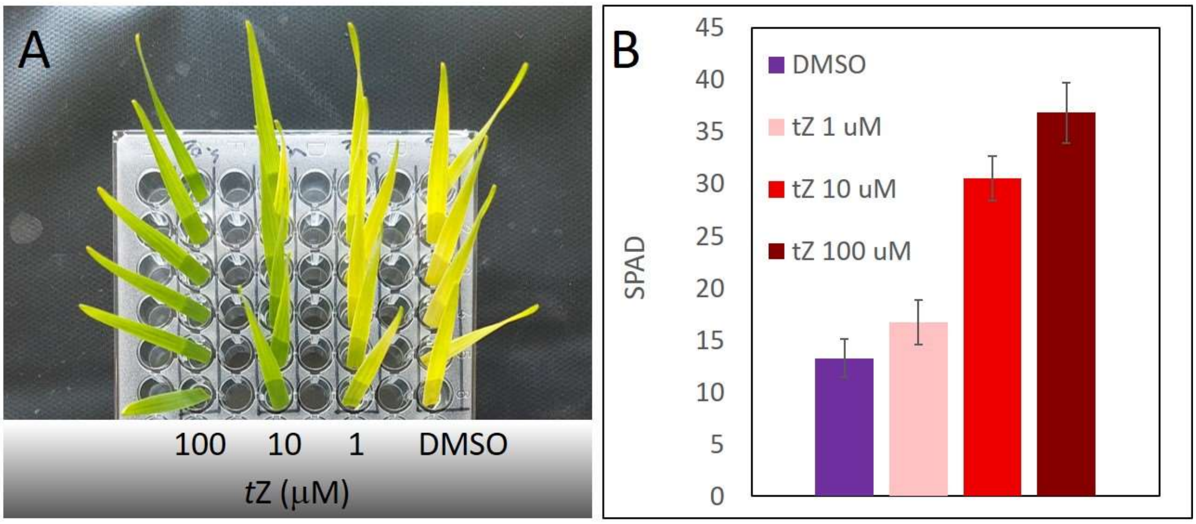

Table S1 Endogenous levels of individual cytokinin forms (pmol g^{-1} FW) in intact wheat leaves. Lowercase letters indicate statistically significant differences based on the two-tailed Student's *t*-test (*a*, *b*, and *c* correspond to *P*-values of $0.05 > P > 0.01$, $0.01 > P > 0.001$, and $P < 0.001$, respectively); watered control *vs* drought control (black letters), drought control *vs* drought + MTU (red letters). *<LOD* denotes a value below the limit of detection.

	Watered control	Drought control		Drought + MTU		
Total CKs	**141.22 ± 20.86**	**363.84 ± 39.72**	***c***	**280.16 ± 73.17**	***a***	
Bases	13.28 ± 4.03	15.53 ± 5.59		7.79 ± 4.09		
Ribosides	2.37 ± 0.42	4.31 ± 1.03	*a*	2.69 ± 0.80		
Nucleotides	0.30 ± 0.03	9.74 ± 1.07	*c*	5.75 ± 2.63	*a*	*a*
O-glucosides	118.22 ± 20.92	301.23 ± 37.84	*c*	243.84 ± 57.27	*a*	
N-glucosides	7.05 ± 1.20	33.03 ± 8.59	*b*	20.10 ± 9.58		
tZ	0.12 ± 0.01	0.32 ± 0.03	*c*	0.20 ± 0.08		
tZR	0.01 ± 0.00	0.05 ± 0.03		0.04 ± 0.04		
tZOG	0.22 ± 0.03	0.59 ± 0.10	*c*	0.47 ± 0.23		
tZROG	*<LOD*	*<LOD*		*<LOD*		
tZ7G	*<LOD*	*<LOD*		*<LOD*		
tZ9G	5.15 ± 0.83	24.12 ± 5.50	*b*	14.91 ± 9.22		
tZR5'MP	*<LOD*	*<LOD*		*<LOD*		
**Total *tZ*-types**	**5.28 ± 0.84**	**24.49 ± 5.48**	***c***	**15.14 ± 9.22**		
cZ	14.87 ± 2.79	16.78 ± 3.64		7.50 ± 4.07	*a*	*a*
cZR	1.98 ± 0.39	3.64 ± 0.93	*a*	2.05 ± 0.90		
cZOG	108.13 ± 20.99	254.87 ± 28.80	*c*	206.41 ± 47.34	*a*	
cZROG	9.87 ± 0.99	45.77 ± 13.51	*b*	36.96 ± 10.53	*b*	
cZ9G	0.69 ± 0.13	2.87 ± 0.89	*b*	5.76 ± 2.08	*a*	
cZR5'MP	<LOD	3.30 ± 8.99	*c*	0.90 ± 2.28	*c*	*a*
**Total *cZ*-types**	**131.60 ± 20.87**	**327.47 ± 37.20**	***c***	**258.39 ± 63.92**	***a***	
DHZ	*<LOD*	*<LOD*		*<LOD*		
DHZR	0.01 ± 0.01	0.01 ± 0.00		0.01 ± 0.00		
DHZOG	*<LOD*	*<LOD*		*<LOD*		
DHZROG	*<LOD*	*<LOD*		*<LOD*		
DHZ7G	*<LOD*	*<LOD*		*<LOD*		
DHZ9G	*<LOD*	*<LOD*		*<LOD*		
DHZR5'MP	*<LOD*	*<LOD*		*<LOD*		
**Total DHZ-types**	**0.01 ± 0.01**	**0.01 ± 0.00**		**0.01 ± 0.00**		
iP	0.25 ± 0.08	0.24 ± 0.07		0.09 ± 0.03	*a*	*a*
iPR	0.37 ± 0.10	0.61 ± 0.13	*a*	0.59 ± 0.24		
iP7G	*<LOD*	*<LOD*		*<LOD*		
iP9G	1.21 ± 0.31	6.04 ± 2.26	*a*	2.91 ± 0.87	*a*	
iPR5'MP	0.30 ± 0.03	0.75 ± 0.21	*b*	0.50 ± 0.07	*b*	
**Total iP-type**	**2.13 ± 0.33**	**7.64 ± 2.47**	***b***	**4.10 ± 0.07**	***a***	

Table S2 Endogenous levels of brassinosteroids (ng g^{-1} FW) in intact wheat leaves. Values are means ± SD of three biological replicates (each analysed in two technical replicates). Lowercase letters show statistically significant differences based on the two-tailed Student's *t*-test (*a*, *b*, and *c* correspond to *P*-values of $0.05 > P > 0.01$, $0.01 > P > 0.001$, and $P < 0.001$, respectively); watered control *vs* drought control (black letters), drought control *vs* drought + MTU (red letters); - means not significantly different. Of the brassinosteroids analysed brassinolide, castasterone, teasterone, 28-homobrassinolide, dolichosterone, homodolichosterone, dolicholide, homodolicholide and typhasterol were not detected in any sample. Bioactive brassinosteroids are marked in blue.

Treatment	*24-epibrassinolide*	*24-epicastasterone*	*28-norcastasterone*	*norteasterone*	*homocastasterone*	*28-norbrassinolide*	Total
Watered control	1.86 ± 0.27	0.36 ± 0.16	1.38 ± 0.07	0.35 ± 0.09	12.16 ± 1.83	1.69 ± 0.06	**17.8 ± 2.4**
Drought control	6.39 ± 1.42 ***a***	1.5 ± 0.4	7.08 ± 0.65 ***c***	0.54 ± 0.13	20.14 ± 3.81	2.46 ± 0.74	**38.1 ± 3.5 *b***
Drought + MTU	3.25 ± 1.29 **-, *a***	0.06 ± 0.01 ***b*, -**	8.77 ± 0.59 ***c*, *b***	0.34 ± 0.08 **-, *b***	13.76 ± 3.00 **-, *b***	1.69 ± 0.34	**27.9 ± 2.1 *a*, *b***

Table S3 Endogenous levels of gibberellins (ng g^{-1} FW) in intact wheat leaves. Values are means ± SD of three biological replicates, each of which was analysed in two technical replicates. There are no significant differences between any samples at $P < 0.05$ based on the two-tailed Student's *t*-test. $GA_{12}ald$ and GA_{12} were not estimated. Of the 13-hydroxylated gibberellins, GA_{53}, GA_{20}, GA_{29} and GA_6 were not determined in any sample. Bioactive gibberellins are marked in blue.

Treatment	13-non-hydroxylated gibberellin								
	GA_{15}	GA_{24}	GA_{13}	GA_9	GA_7	GA_4	GA_{51}	GA_{34}	Total
Watered control	1.00 ± 0.26	0.99 ± 1.2	0.09 ± 0.02	18.81 ± 4.95	0.11 ± 0.01	0.80 ± 0.25	3.94 ± 0.90	0.12 ± 0.04	**25.9 ± 5.3**
Drought control	4.89 ± 4.13	1.01 ± 0.11	0.18 ± 0.05	21.24 ± 7.06	0.09 ± 0.00	0.99 ± 0.46	6.62 ± 2.6	0.10 ± 0.05	**35.1 ± 13.1**
Drought + MTU	1.45 ± 0.20	1.10 ± 0.38	0.24 ± 0.04	24.43 ± 7.96	0.09 ± 0.04	0.90 ± 0.05	12.09 ± 4.26	0.11 ± 0.07	**40.4 ± 11.9**

Treatment	13-hydroxylated gibberellin						
	GA_{19}	GA_{44}	GA_1	GA_8	GA_5	GA_3	Total
Watered control	0.77 ± 0.25	0.76 ± 0.21	0.46 ± 0.11	0.34 ± 0.03	0.20 ± 0.11	1.02 ± 0.16	**3.6 ± 0.3**
Drought control	0.96 ± 0.29	0.98 ± 0.41	0.44 ± 0.12	0.21 ± 0.03	0.16 ± 0.06	0.93 ± 0.07	**3.7 ± 0.5**
Drought + MTU	1.47 ± 0.83	1.25 ± 0.34	0.89 ± 0.34	0.23 ± 0.10	0.16 ± 0.02	0.90 ± 0.28	**4.9 ± 0.6**

Table S4. List of primers used in this work and corresponding references.

Gene	GenBank accession no.	primers	sequences	Publication
TaEF1-b	EU022331	HvEF2_fw	CCGCACTGTCATGAGCAAGT	This work
		HvEF2_rev	GGGCGAGCTTCCATGTAAAG	
TaGAPDH	D13147	TaGAPDH_fw	TTAGACTTGCGAAGCCAGCA	Zhang *et al.*, 2012
		TaGAPDH_rev	AAATGCCCTTGAGGTTTCCC	
TaIPT5	NP001121194	TaIPT5_fw	CACAGACAAGCTGCACGAAGGA	Song *et al.*, 2012
		TaIPT5_rev	CGATCGGTCAGCTTGTGTACCAAC	
TaIPT8	ABY78887	TaIPT8_fw	GACGTGGAGGAGGCGCTCCT	Song *et al.*, 2012
		TaIPT8_rev	ATGCGCCGGATCTTGGACA	
TaCKX3	JN128585	TaCKX3_fw	CGTGGCTCAACCTCTTCGTC	Ma *et al.*, 2011
		TaCKX3_rev	GTTCGGGTCCCACTTGCTC	
TaCKX4	JN128586	TaCKX4_fw	TGCTGTCTCGGCTGAGATACATACAG	Song *et al.*, 2012
		TaCKX4_rev	TGACGTCCTGTTGTCCCACTTTG	
TaCKX6	JN128587	TaCKX6_fw	CGACGAGATCTTACGGTTCT	Zhang *et al.*, 2012
		TaCKX6_rev	GACCGATGGATCAGCCA	
TaGLU1	JN128601	TaGLU1_fw	AACTTCGAATGGAGCCTCGG	This work
		TaGLU1_rev	GCTGTCATGGTTGCGTTGTT	
TaGLU2	JN128600	TaGLU2_fw	GCTCTTCAACATCCCGCTCT	This work
		TaGLU2_rev	AGTCGGCGAGTTCCACAAAT	
TaRR1	JN128605	TaRR1_fw	GGCTACGACCTCCTCAAAGCCA	Song *et al.*, 2012
		TaRR1_rev	GCGGCACATCCTTGCTCTGA	
TaRR4	JN128606	TaRR4_fw	AGGAGGTGGGCGTGAATTTGA	Song *et al.*, 2012
		TaRR4_rev	TGCGACTTGAGCTTCTTCATGTCA	
TaRR9	JN128608	TaRR9_fw	CGTGGATGACAGCGTCCTTGA	Song *et al.*, 2012
		TaRR9_rev	CTTCCTCCAGGCATCTGTTGATTC	
TaSAG3	AB539591	TaSAG3_fw	TGTTCTTGACGACGATGGTG	Kajimura *et al.*,
		TaSAG3_rev	TGAGCACTAAGCGCAGCA	2010
TaSAG5	CJ699015	TaSAG5_fw	GGCAAGGGGATGAGAATAG	Kajimura *et al.*,
		TaSAG5_rev	CTTCTGATGCCTTCTTTGT	2010
TaSAG8	AB539589	TaSAG8_fw	CACCTCCTCCCCTCCGCATC	Kajimura *et al.*,
		TaSAG8_rev	TTGATTCCAGTTTTGTCCAG	2010
TaCAT	GU984379	TaCAT_fw	GAGCCGTCTCAGCTCCAAG	This work
		TaCAT_rev	ACGGCCCTGAAGCAGATTTC	
TaSOD	JN257665	TaSOD_fw	CAGAGGGTGCTGCTTTACAA	Baek *et al.*, 2004
		TaSOD_rev	GGTCACAAGAGGGTCCTGAT	

Baek KW, Skinner DZ. 2004. Quantitative real-time PCR method to detect changes in specific transcript and total RNA amounts. *Electronic Journal of Biotechnology* **7**:55–60.

Ma X, Feng DS, Wang HG, Li XF, Kong LR. 2011. Cloning and expression analysis of wheat cytokinin oxidase/dehydrogenase gene TaCKX3. *Plant Molecular Biology Reporter* **29**:98–105.

Song J, Jiang L, Jameson PE. 2012. Co-ordinate regulation of cytokinin gene family members during flag leaf and reproductive development in wheat. *BMC Plant Biology* **12**:78–95.

Zhang L, Zhao YL, Gao LF, Zhao GY, Zhou RH, Zhang BS, Jia JZ. 2012. TaCKX6-D1, the ortholog of rice OsCKX2, is associated with grain weight in hexaploid wheat. *New Phytologist* **195**:574–584.

Table S5. The effect of MTU on yield and yield-forming parameters in field-grown winter wheat and spring barley. Values are means of five or six biological replicates ± SD. Asterisks indicate statistically significant differences determined by the two-tailed Student's *t*-test at *P*-values of 0.15 > *P*.

Country/ year	Treatment	Appl. timing (BBCH)	Yield (t ha^{-1})	% of control	TGW (g)	% of control
		**WINTER WHEAT**				
CZ 2015	control	-	11.422 ± 0.45	100 %	50.28	100 %
	MTU	seed coat.	11.503 ± 0.85	+ 0.7 %	49.74	- 1.1 %
	MTU	25	12.323 ± 0.75 *	+ 7.9 %	49.56	- 1.4 %
CZ 2016	control	-	9.521 ± 0.28	100 %	53.08	100 %
	MTU	seed coat.	9.319 ± 0.68	- 2.1 %	55.11	+ 3.9 %
	MTU	25	10.175 ± 0.85 *	+ 6.9 %	52.21	- 1.6 %
CZ 2017	control	-	12.797 ± 0.57	100 %	51.34	100 %
	MTU	seed coat.	13.414 ± 0.51 *	+ 4.8 %	52.56	+ 2.4 %
	MTU	25	13.596 ± 0.58 *	+ 6.2 %	53.54	+ 4.3 %
	MTU	51	13.454 ± 0.36 *	+ 5.1 %	52.79	+ 2.8 %
Mean		***seed coat.***	**-**	***+ 1.1 %***	**-**	***+ 1.7 %***
Mean		***25***	**-**	***+ 7.0 %***	**-**	***+ 0.4 %***
UK 2019	Control	-	9.986 ± 0.53	100 %	46.45	100
	MTU	32	10.225 ± 0.41	+ 2.4 %	42.45	- 8.6 %
	MTU	39	10.622 ± 0.62 *	+ 6.4 %	44.75	- 3.7 %
	MTU	50-55	10.241 ± 0.67	+ 2.6 %	44.33	- 4.6 %
UK 2020†	Control	-	9.890 ± 0.54	100 %	-	-
	MTU	32	10.227 ± 0.65	+ 3.3 %	-	-
	MTU	39	10.625 ± 0.44 *	+ 7.4 %	-	-
	MTU	55	10.243 ± 0.68	+ 3.5 %	-	-
UK 2020†	Control	-	9.925 ± 0.63	100 %	-	-
	MTU	32	10.113 ± 0.42	+ 1.9 %	-	-
	MTU	39	10.054 ± 0.52	+ 1.3 %	-	-
	MTU	55	10.254 ± 0.41	+ 3.3 %	-	-
Mean		***32***	-	***+ 2.5 %***	-	-
Mean		***39***	-	***+ 5.0 %***	-	-
Mean		***55***	-	***+ 3.1 %***	-	-

Country/ year	Treatment	Appl. timing (BBCH)	Yield (t ha^{-1})	% of control	TGW (g)	% of control
		**SPRING BARLEY**				
CZ 2014	control	-	7.653 ± 1.59	100 %	45.85	100 %
	MTU	23-25	8.517 ± 0.72	+ 11.3 %	47.18	+ 2.9 %
	MTU	51-53	7.486 ± 0.53	- 2.2 %	46.77	+ 2.0 %
CZ 2015†	control	-	10.854 ± 0.56	100 %	50.98	100 %
	MTU	23-25	11.368 ± 0.82	+ 4.7 %	51.53	+ 1.1 %

	MTU	51-53	10.970 ± 0.49	+ 1.1 %	50,93	- 0.1 %
CZ 2015†	control	-	7.016 ± 0.58	100 %	46.07	100 %
	MTU	23-25	7.585 ± 0.12 *	+ 8.1 %	47.14	+ 2.3 %
	MTU	51-53	7.425 ± 0.79	+ 5.8 %	47.68	+ 3.5 %
CZ 2016	control	-	7.637 ± 0.65	100 %	46.21	100 %
	MTU	23-25	8.246 ± 0.64 *	+ 8.0 %	46.02	- 0.4 %
	MTU	51	7.380 ± 0.70	- 3.4 %	46.64	+ 0.9 %
Mean		***21 – 25***	***-***	***+ 8.0 %***	***-***	***+ 2.1 %***
Mean		***51 – 53***	***-***	***+ 1.3 %***	***-***	***+ 1.2 %***

TGW – thousand-grain weight, † - the experiment was done at two different locations in the same region

Table S6. Endogenous levels of individual cytokinin groups and total cytokinin content (pmol g^{-1} FW) in intact barley (*Hordeum vulgare* L.) leaves. Plants were harvested and cytokinin content was analyzed at the tillering stage (BBCH 25-27). The analysis was performed as described in the Material and methods section. MTU (5 μM) was applied to foliage by spraying 14 days prior to analysis. Values are means ± SD of four biological replicates. Asterisks indicate statistically significant differences (at $P < 0.05$) based on the two-tailed Student's *t*-test.

	Control	MTU	
Total CKs	119.39 ± 33.88	74.47 ± 5.40	*
Bases	1.13 ± 0.19	1.66 ± 0.36	
Ribosides	3.05 ± 0.48	4.43 ± 0.76	
Nucleotides	0.47 ± 0.09	0.63 ± 0.16	
O-glucosides	112.36 ± 33.38	64.93 ± 4.76	*
N-glucosides	2.38 ± 0.33	2.82 ± 0.33	

Data S1 Eco-toxicity test results for MTU. MTU was tested at concentrations of 0.0005 - 0.016 kg ha^{-1}. All tests gave the same result - TOXICITY NOT DETECTED.

FC test – Determination of the effect of MTU on the reproduction of *Folsomia candida (Collembola),* a "standard" soil arthropod.

EC test – Determination of the effect of MTU on the reproduction of *Enchytraeus crypticus,* a common soil invertebrate and model species in soil ecotoxicology.

SNA test – Determination of inhibition of short-term nitrifying activity.

In these tests, the values of **EC20** (Effect concentration – effective concentration at which a 20 % inhibitory effect occurred), **EC50** (Effect concentration – effective concentration at which a 50 % inhibitory effect occurred), **LOEC** (Lowest observed effect concentration – lowest concentration at which an adverse reaction was observed) and **NOEC** (No observed effect concentration – highest concentration at which no adverse effect was observed) were measured.

OECD-C test - Determination of substrate-induced inhibition of respiration by the soil microbial community in a long-term incubation experiment. The OECD-C test is performed at the recommended dose, twice the recommended dose, and under control conditions. In all cases, the degree of respiratory inhibition was below 25 % on day 28.
